# Gastrointestinal Transit Times in Health as Determined Using Ingestible Capsule Systems: A Systematic Review

**DOI:** 10.3390/jcm12165272

**Published:** 2023-08-13

**Authors:** Gursharan Kaur Nandhra, Phakanant Chaichanavichkij, Malcolm Birch, S. Mark Scott

**Affiliations:** 1 National Bowel Research Centre and GI Physiology Unit, Blizard Institute, Centre for Neuroscience, Surgery & Trauma, Queen Mary University of London, London E1 4NS, UK; p.chaichanavichkij@qmul.ac.uk (P.C.); m.j.birch@qmul.ac.uk (M.B.); m.scott@qmul.ac.uk (S.M.S.); 2 Clinical Physics, Barts Health NHS Trust, The Royal London Hospital, London E1 2BL, UK

**Keywords:** gastrointestinal, ingestible capsule, motility, transit time

## Abstract

Background: Ingestible capsule (IC) systems can assess gastrointestinal (GI) transit times as a surrogate for gut motility for extended periods of time within a minimally invasive, radiation-free and ambulatory setting. Methods: A literature review of IC systems and a systematic review of studies utilizing IC systems to measure GI transit times in healthy volunteers was performed. Screening for eligible studies, data extraction and bias assessments was performed by two reviewers. A narrative synthesis of the results was performed. Results: The literature review identified 23 different IC systems. The systematic review found 6892 records, of which 22 studies were eligible. GI transit time data were available from a total of 1885 healthy volunteers. Overall, seventeen included studies reported gastric emptying time (GET) and small intestinal transit time (SITT). Colonic transit time (CTT) was reported in nine studies and whole gut transit time (WGTT) was reported in eleven studies. GI transit times in the included studies ranged between 0.4 and 15.3 h for GET, 3.3–7 h for SITT, 15.9–28.9 h for CTT and 23.0–37.4 h for WGTT. GI transit times, notably GET, were influenced by the study protocol. Conclusions: This review provides an up-to-date overview of IC systems and reference ranges for GI transit times. It also highlights the need to standardise protocols to differentiate between normal and pathological function.

## 1. Introduction

Ingestible capsule (IC) systems have been used to measure gastrointestinal (GI) function since the late 1950s [1,2]. Subsequent advances in microelectronics, signal processing, material and data science have led to a revolution in the development of “wearable” technology, which has recently revived the use of IC systems for the assessment of GI motility [3]. For a GI motility test to be useful, it must make measurements that are reflective of GI function and have the ability to differentiate between normal and pathological. One primary functional measure of GI motility is gut transit times, which provides an indication of the duration it takes ingested content to travel from one segment to the next (i.e., stomach, small intestine or colon) or within different regions of the same organ (i.e., right, left or rectosigmoid colon transit). Transit time measurements are important as, firstly, they are an objective measure, and secondly, dysmotility can either cause delayed or accelerated transit of ingested content within one or more regions, which makes it a potential clinical biomarker. The continuous measurement of whole-gut and segmental transit times has been made possible by IC systems, which have several advantages over conventional methods, such as being minimally invasive, radiation-free and ambulatory, thereby providing an assessment of the study subject under near-physiological conditions [4].

Establishing robust normative reference values for whole-gut or segmental transit times in healthy volunteers is key to the application of any GI motility test, and although these have been reported for individual IC systems [5,6], to the best of our knowledge, a systematic review comparing the values between different systems has yet to be performed. Therefore, our primary objectives were to undertake:

1. a literature review of IC systems used clinically and in research for the assessment of GI motility;
2. a systematic review of studies utilising IC systems to measure and report gastric emptying time (GET), small intestinal transit time (SITT), colonic transit time (CTT) and whole-gut transit time (WGTT) in healthy volunteers.

A secondary objective of the systematic review was to evaluate the influence of the study protocol on the transit time estimates.

## 2. Materials and Methods

### 2.1. Protocol and Registration

The protocol for this systematic review was registered on PROSPERO (www.crd.york.ac.uk/PROSPERO (accessed on 8 October 2021): registration number CRD42021271289). The review was conducted in line with the protocol and reported in accordance with the Preferred Reporting Items for Systematic Reviews and Meta-Analyses (PRISMA) guidelines [7].

### 2.2. Eligibility Criteria

The study eligibility criteria, in terms of patients, interventions, comparators, outcomes and study design (PICOS), are provided in Table 1.

### 2.3. Information Sources and Search Strategy

PubMed and EMBASE were used to search for eligible English-language studies. There were no restrictions on the study publication date. The search end date was 3 February 2023. The reference lists of included studies were reviewed for any additional eligible studies. The full search strategy consisted of three queries, as outlined in Supplementary Materials (Section S1, Table S1).

### 2.4. Study Selection

The search was performed by one author (P.C.), who extracted all the results into a spreadsheet (Microsoft Excel, Office 365 ProPlus 2016, Microsoft Corporation, Redmond, WA, USA). After the removal of duplicate records, the remaining study titles and abstracts were independently screened by two authors (G.K.N. and P.C.) against the eligibility criteria. Full-text articles of potentially eligible studies were retrieved and reviewed independently by two authors (G.K.N. and P.C.) with any disagreements resolved by the senior author (S.M.S.).

### 2.5. Data Collection, Data Items and Summary Measures

Study characteristics and outcome data were extracted by G.K.N. and verified by P.C. The following data were extracted from the eligible studies: publication year, country of origin, study type, healthy volunteer sample size and final sample size (due to drop-outs, incomplete datasets etc.), number of female subjects, mean or median age of subjects, type of IC system used, any bowel preparation procedures performed prior to capsule ingestion, capsule ingestion protocol (i.e., with or without meal) where available, the ingestion meal calorie content, fasting duration following capsule ingestion and GI transit times.

### 2.6. Assessment of Risk of Bias

Risk of bias was assessed using the National Heart, Lung and Blood Institute (NHLBI) quality assessment tools for studies (https://www.nhlbi.nih.gov/health-topics/study-quality-assessment-tools (accessed on 23 February 2023)). The assessment was carried out by two reviewers (G.K.N. and P.C.) independently, with any disagreements discussed and resolved with the senior author (S.M.S.). Depending on the tool used, each study was assessed against a series of questions which required reviewers to answer “yes”, “no” or “cannot determine/not applicable/not reported”. Any “yes” answers scored 1 point, whereas “no” or “cannot determine/not reported” scored 0, thereby indicating a potential flaw in the study. The quality of a study was graded on the percentage of points scored such that a score between 0% and 33% indicated a high risk of bias, ≥34–66% indicated a moderate risk of bias and ≥67% indicated a low risk of bias.

### 2.7. Synthesis of Results

All extracted GI transit times were converted into hours, with 95% confidence intervals (CI) calculated for studies that reported means and standard deviations. All estimates of transit times, regardless of whether the summary statistics were reported as means or medians, were presented in bubble charts, with the study reference on the x-axis, the transit time estimate on the y-axis and the size of the bubble representing the sample size. The 95% CI for the mean estimates of transit times were presented as error bars, and for studies reporting medians, the interquartile ranges or 95% confidence intervals for medians were used, where available. Where studies reported multiple results due to the ingestion of multiple capsules or variations in the protocol, the results were superimposed vertically in the bubble charts to indicate that the results were from the same study. The effect of the study protocol on transit times was illustrated in swarm plots. Due to heterogeneity in the design of the eligible studies and differences in the inclusion criteria, pooling of the results was not considered to be appropriate. Therefore, a meta-analysis was not performed. A narrative synthesis was carried out instead.

## 3. Results

### 3.1. Results of the Literature Review

The literature review identified 23 different IC systems, which were grouped into 5 categories: (1) intraluminal imaging systems; (2) pH, temperature and pressure-sensing systems; (3) single-sensor temperature-sensing systems; (4) magnetic tracking systems; and (5) gas sensing systems—see Table 2 for details. A summary of each system category is provided in the Supplementary Materials (Section S2).

### 3.2. Study Selection for the Systematic Review

**Figure 1:**
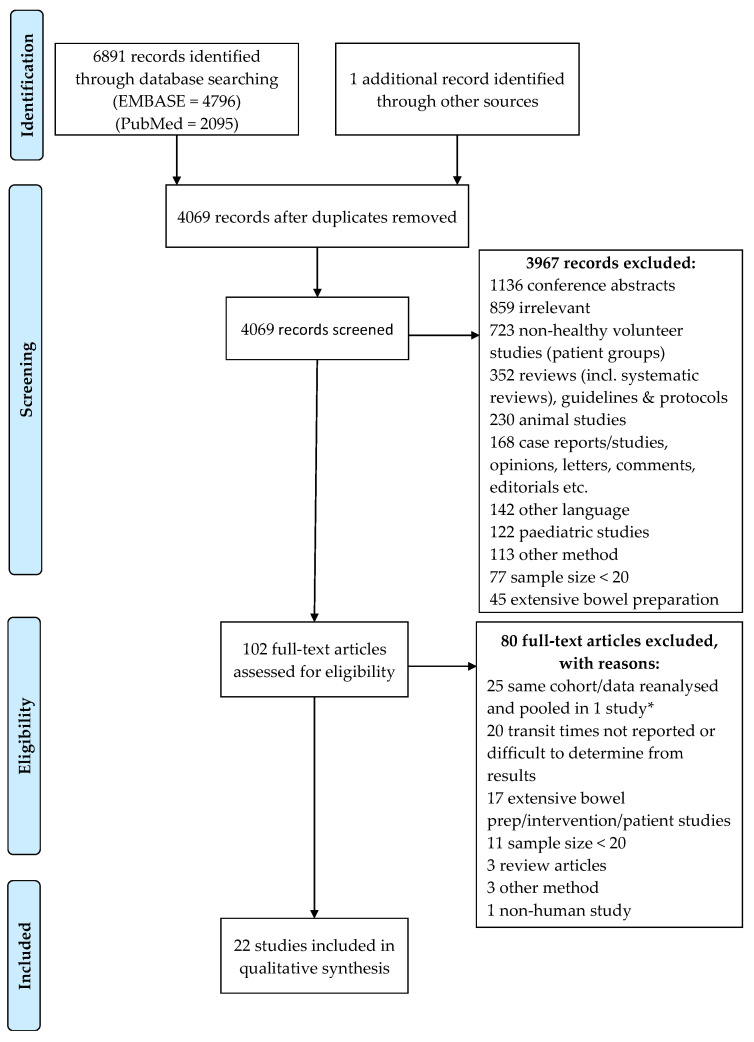
PRISMA flow diagram. * For a list of studies, see Section S3 in Supplementary Material.

The study search and selection process are described in the PRISMA flow diagram (Figure 1). A total of 6892 records were identified from electronic and manual searches. After duplicate removal, 4069 records were screened, of which 102 full text articles were reviewed. Eighty of these did not meet the eligibility criteria and were therefore excluded. Twenty-two articles were thus found to meet the inclusion criteria, twenty-one of which were identified from database searches and one [63] was identified from a manual screening of the references. Several individual, potentially eligible studies were excluded from the analysis as their data had been reanalysed and pooled in other single studies (see Section S3 in Supplementary Materials).

Three studies [64,65,66] were conducted at the same centre. The lead author was contacted to check whether the studies had reused published data. No response was received; however, it was noted that the study periods did not overlap, so all three studies were included in the review. Capsule 1 data from Haase et al. [67] were pooled in one study [5]; these data were therefore excluded from the analysis. Mark et al. [68] presented the transit time data in a box plot. The study authors were contacted by email for the raw data, which were provided, so the summary statistics (medians and interquartile ranges) of the GI transit times were calculated.

### 3.3. Characteristics of Included Studies

The characteristics of the included studies are provided in Table 3. The eligible studies were published between 1988 and 2022. Based on the NHLBI quality assessment study classifications, eight studies [5,6,61,64,65,66,69,70] were classed as observational cohort studies, seven [30,55,67,71,72,73,74] were case series studies, five [63,68,75,76,77] were randomised controlled trials, one [78] was a before-after (pre-post) study and one [79] was a case-control study. Fourteen of the twenty-two studies were from single centres, eight of which originated from the EU, two from Japan, one from Switzerland, two from the United Kingdom (UK) and one from the United States of America (USA). The remaining seven studies were multicentre studies (see Table 3). All but five studies [64,65,66,76,79] were exclusively carried out in healthy volunteers. Seventeen studies published new data, whereas the remaining five [5,6,70,78,79] utilised previously published data, which were either from the same cohort or pooled and re-analysed to estimate the transit times. Funding sources were declared in all but seven of the included studies [30,55,68,70,71,75,78], whereas statements on ethical approval were available in all of the studies. A combined total of 1885 healthy volunteers were recruited in the included studies, of which 681 (36%) were female. The sample sizes ranged between 20 and 580 (median 50). Two studies [68,78] were solely carried out in men. The percentage of women included in the remaining twenty studies ranged between 11% and 96% (median 53%). Eleven studies [30,55,63,69,70,73,74,76,77,78,79] reported the subject ages as means (ranging between 21 and 49 years), seven [5,6,61,67,68,71,72] reported them as medians (ranging between 25 and 40 years), three studies [64,65,66] only provided age ranges (ranging between 16 and 66 years) and one study [75] did not specify whether the summary statistic for age was reported as a mean or median (reported value: 35.4 years).

Nine studies utilised imaging systems, seven used pH-sensing systems, four used magnetic/electromagnetic system, one study used a single-sensor temperature sensing system and one study used the gas-sensing system alongside a pH sensing system. All but two of the studies utilised one capsule per subject; Haase et al. [67] utilised three capsules per subject and Thwaites et al. [61] utilised two capsules per subject (a gas-sensing and pH sensing capsule). A review of the study protocols revealed that the subjects fasted overnight before capsule ingestion in all but three of the studies, [69,70,76], which did not include any statements on bowel preparation. Nine studies [63,64,65,66,70,72,75,76,78] did not include any statements on the ingestion protocol, i.e., whether capsules were ingested with a meal or not. The remaining ten studies stated that the capsule was either ingested with a liquid [30,55,69,71,74] or with a meal [5,6,54,61,67,68,77,79]. Six of the seven studies where subjects ingested the capsule with a meal provided the total caloric intake of the ingestion meal (ranging between 255 kcal and 964 kcal); the authors of the seventh study [67] were contacted for the ingestion meal calorific content, and this was provided, ranging between 354 kcal for female subjects and 602 kcal for male subjects.

Fourteen (64%) studies required the subjects to ingest capsules in the morning. One study [69] required the subjects to ingest the capsule in the afternoon. Seven (37%) studies did not specify a capsule ingestion time. The study by Haase et al. [67], which utilised multiple capsules, required the subjects to ingest the first capsule in the morning, the second in the evening and the third capsule on the following day in the morning. As for the fasting duration following capsule ingestion, this was specified in twelve studies [5,6,55,61,67,68,69,71,72,74,77,79] and varied between 2.5 h to 6 h or until capsule passage into the duodenum was seen. In four studies, the subjects ingested water [73] or a liquid meal [64,65,66] within 45 mins to 60 mins of capsule ingestion. Six studies [30,63,70,75,76,78] did not specify whether the subjects fasted following capsule ingestion.

**Table 3:** Summary of studies reporting GI transit times in healthy populations using IC systems.

Author	Publication Year	Country	Study Design *	ITT HV Sample Size (N)	Female (N)	Actual HV Sample Size (N)	Age (Years)	Min	ICS Sensor Type	Bowel Prep	Capsule Ingested with Meal?	Ingestion Meal kCal	Capsule Ingestion Time	Fasting Duration after Capsule Ingestion (Hours)	GET	SITT	CTT	WGTT
Evans [71]	1988	UK	Case series	72	21	66 (SITT) 32 (WGTT)	Median: 26 Range: 20–83	RTC	pH	Overnight fast	No—only water	None	Morning (08:30)	Subjects fasted until capsule left stomach (indicated by pH rise)		✓		✓
Fallingborg [72]	1989	Denmark	Case series	39	21	39	Median: 33 Range: 18–65	RTC	pH	Overnight fast	Not specified	Not specified	Morning (~08:00)	Subjects fasted until capsule left stomach as indicated by pH rise	✓			
Goldstein [63] †	2007	Multicentre (USA, Israel)	Randomised controlled trial	580	66	113†	Mean: 32.2 ± 10.0 Range: 18–65	PillCam SB	Imaging	12 h fast	Not specified	Not specified	Not specified	Not specified	✓			
Malagelada [64]	2008	Spain	Observational cohort study	50	27	34	Range: 18–36	PillCam SB	Imaging	Overnight fast	Not specified	None	Not specified	Liquid meal (300 mL (1 kCal/mL)) ingested 60 min after capsule ingestion.		✓		
Hocke [30]	2009	Germany	Case series	21	10	21	Female mean 35.8 ± 11.6 Male mean 40.4 ± 13.6	3D-MAGMA	Magnetic	Overnight fast	No—only water	None	Morning (between 08:00 and 12:00)	Not specified	✓			
Hooks [75]	2009	USA	Randomised controlled trial	40	13	20 (GET) † 19 (SITT) †	35.4	PillCam SB	Imaging	8 h fast	Not specified	Not specified	Not specified	Not specified	✓	✓		
Fujimori [78]	2010	Japan	Before-after study with no control group	55	0	55	Mean 37 ± 8	PillCam SB	Imaging	12 h fast	Not specified	Not specified	Not specified	Not specified	✓	✓		
Malagelada [65]	2012	Spain	Observational cohort study	70	39	52	Range: 18–66	PillCam SB	Imaging	Overnight fast	Not specified	None	Morning	Liquid meal ingested (300 mL 1 kCal/mL) 45 min after capsule ingestion.	✓	✓		
van der Schaar [55]	2013	Multicentre (The Netherlands, USA)	Case series	20	14	20	Study 1 mean: 21.6 Study 2 mean: 20.6 Range: 19–25	IntelliCap	pH and temperature	Overnight fast	No—only water	None	Morning	4 h	✓	✓	✓	✓
Haase [67]	2014	Multicentre (Denmark, Switzerland, UK, Czech Republic)	Case series	20	10	Capsule 1: 20 Capsule 2: 19 (GET and SITT) 17 (CTT) 17 (WGTT) Capsule 3: 17 (GET) 18 (SITT) 15 (CTT and WGTT)	Median: 32 Range: 26–52	3D-Transit	Electromagnetic	Overnight fast	Yes	354 kCal for female subjects 602 kCal for male subjects.	Capsule 1: Day 1 morning Capsule 2: Day 1 evening Capsule 3: Day 2 morning	6 h	✓	✓	✓	✓
Koziolek [54]	2015	Germany	Case series	20	11	19	Mean: 26.0 ± 4.1 Range: 21–34	WMC	pH, temperature and pressure	At least 10 h fast	Yes	964 kCal	Morning	100 mL water 1, 2, 3 and 4 h after capsule ingestion. Lunch served 4.5 h after capsule ingestion (1000 kCal)	✓			
Malagelada [66]	2015	Spain	Observational cohort study	136	75	132	Range: 16–65	PillCam SB	Imaging	Overnight fast	Not specified	None	Morning	Liquid meal ingested (300 mL 1 kCal/mL) 45 min after capsule ingestion.		✓		
Wang [6]	2015	Multicentre (UK, Sweden, USA)	Observational cohort study	215	87	199 (GET, SITT) 182 (CTT) 194 (WGTT)	Median: 33 Range: 23–49	WMC	pH, temperature and pressure	Overnight fast	Yes	Between 255 kCal and 262 kCal	Morning	6 h	✓	✓	✓	✓
Jianqin [76] ‡	2016	Multicentre (China, Australia, New Zealand)	Randomised controlled trial	45	24	40 ‡	Mean: 46.6 ± 14	OMOM	Imaging	Not specified	Not specified	Not specified	Not specified	Not specified		✓	✓	✓
Monnard [69]	2017	Switzerland	Observational cohort study	27	18	21	Mean: 25 ± 6	CorTemp	Temperature	Not specified	No—only water	None	Afternoon (between 16:00 and 18:00)	Evening meal consumed 2.5–4 h after capsule ingestion				✓
Sakurai [70]	2018	Japan	Observational cohort study	150	74	148	Mean: 48.8 ± 6.5	PillCam SB	Imaging	Not specified	Not specified	Not specified	Not specified	Not specified	✓	✓		
Nandhra [5]	2020	Multicentre (UK, Denmark, Australia, Switzerland)	Observational cohort study	111	58	104 (GET) 111 (SITT, CTT, WGTT)	Median: 40 Range: 21–88	3D-Transit	Electromagnetic	Overnight fast	Yes	Between 255 kCal and 602 kCal	Morning	6 h	✓	✓	✓	✓
O’Grady [74]	2020	Ireland	Case series	71	40	71	Mean: 30.5 ± 6.7 Range: 19–40	PillCam SB	Imaging	Overnight fast	No—only water	None	Morning	4 h	✓	✓		
Mark [68]	2021	Multicentre (Denmark, UK)	Randomised controlled trial	21	0	17 (GET, SITT, CTT) † 18 (WGTT) †	Median: 25 Range: 20–30	3D-Transit	Electromagnetic	Overnight fast	Yes	285 kCal	Not specified	6 h	✓	✓	✓	✓
Sangnes [79] §	2021	Norway	Observational case-control study	26	14	26	Mean: 42 ± 15	WMC	pH, temperature and pressure	Overnight fast	Yes	260 kCal	Morning	6 h	✓	✓	✓	✓
Thwaites [61]	2022	Australia and New Zealand	Observational cohort study	Primary cohort: 26 Validation cohort: 24 Tandem gas-sensing capsule cohort: 20	Primary cohort: 10 Validation cohort: 18 Tandem gas-sensing capsule cohort: 6	Primary cohort: 21–25 Validation cohort: 14–20 Tandem gas-sensing capsule cohort: 17–18	Primary cohort: Median: 35 Range: 31–39 Validation cohort: Median: 25 Range: 23–30 Tandem gas-sensing capsule cohort: Median: 35 Range: 29–39	WMC and Atmo gas sensing capsule	pH, temperature and pressure Gas sensing	Overnight fast	Yes	1092 kJ (260 kCal)	Morning	6 h	✓	✓	✓	✓
Creedon [77] §	2022	UK	Randomised controlled trial	Control group ITT: 26	25	Control group ITT: 14	Control group: Mean: 27.9 ± 5	WMC	pH, temperature and pressure	Overnight fast	Yes	255 kCal	Morning	6 h	✓	✓	✓	✓

CTT—Colonic transit time; GET—Gastric emptying time; ITT—Intention-to-treat; RTC—Radiotelemetry capsule; SITT—Small intestinal transit time; WGTT—Whole-gut transit time; WMC—Wireless motility capsule (SmartPill); * Study design descriptions based on NIHR study quality assessment tools (https://www.nhlbi.nih.gov/health-topics/study-quality-assessment-tools (accessed on 23 February 2023)); † Placebo data used; ‡ Only used data from the overall (N = 40) A2 β-casein arm of the study; § Protocol; for Sangnes et al. [79] specified in von Volkmann et al. [80] and for Creedon et al. [77] specified in Farmer et al. [23].

### 3.4. Risk of Bias and Quality of Included Studies

The results of the risk of bias assessment can be found in the Supplementary Materials, Section S4, Tables S2–S6, with the scores illustrated in Figure S1. Three studies [64,65,72] scored < 33% and were therefore classed as being of low quality with a high risk of bias. Fourteen studies [5,6,30,55,61,66,67,69,70,71,73,76,78,79] scored between 40% and 64% (average 57%), and were therefore considered of moderate quality and at a medium risk of bias. The remaining five studies [63,68,74,75,77] scored ≥ 67% (range: 75–93%; average 84%) and were therefore classed as high-quality studies with a low risk of bias. The average score across all of the studies was 60% (range: 29–93%).

### 3.5. Synthesis of Results

#### 3.5.1. Gastrointestinal Transit Times: Gastric Emptying Time

**Figure 2:**
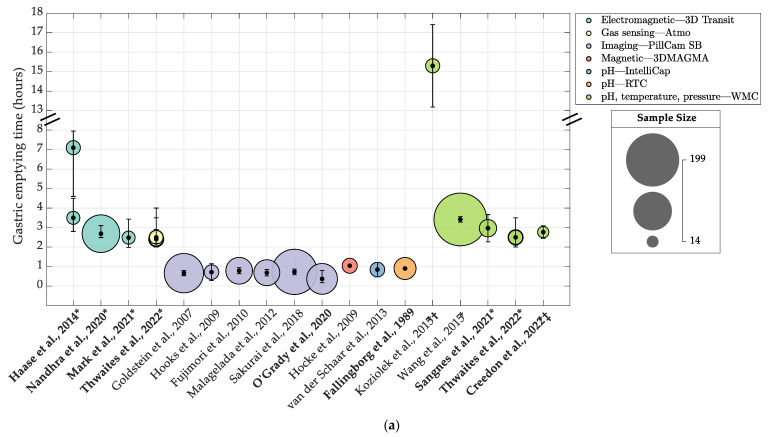

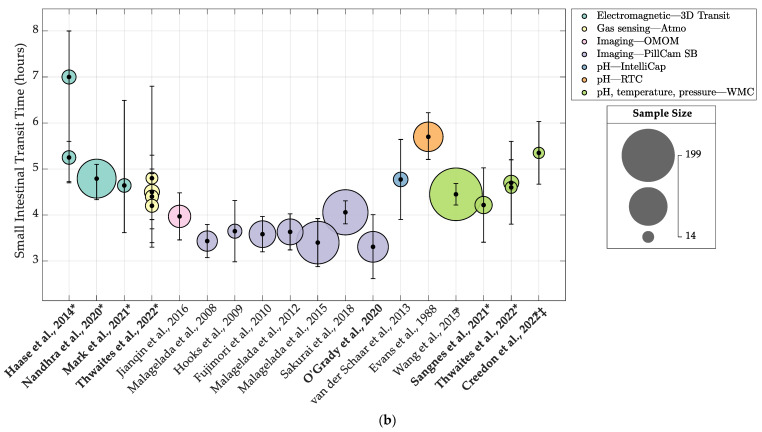

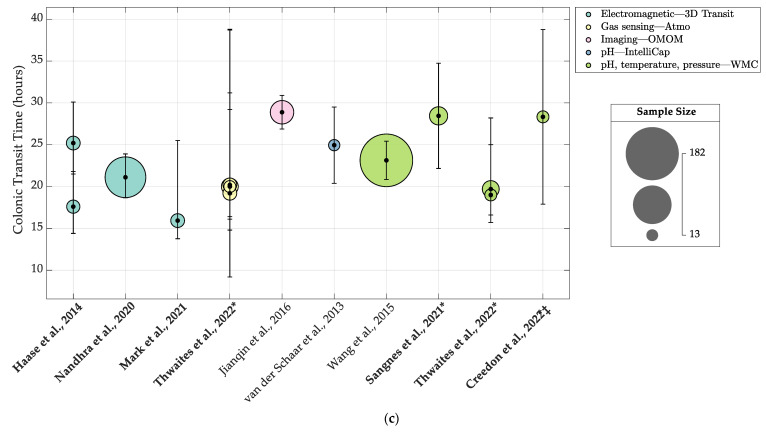

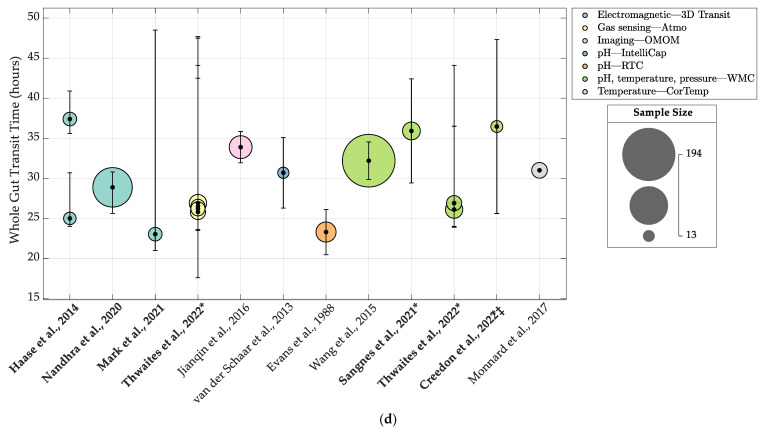
Bubblecharts of studies that reported (**a**) gastric emptying times (GET), (**b**) small intestinal transit time (SITT), (**c**) colonic transit time and (**d**) whole-gut transit time using ingestible capsule systems. Bubbles are colour-coded and grouped by ingestible capsule system type. Values presented are means with error bars representing 95% confidence intervals for all studies except for those in bold type, which reported values as medians. For these studies, error bars represent interquartile ranges or 95% confidence intervals for medians. Study sample size is represented by the bubble size. Some studies reported multiple results due to the use of multiple capsules or different study interventions. These results are superimposed vertically to indicate the results are from the same study [5,6,30,54,55,61,63,64,65,66,67,68,69,70,71,72,74,75,76,77,78,79]. * Studies where the capsule was ingested with a meal. † Study where the capsule was ingested with high-calorie meal. ‡ Only control group intention-to-treat baseline data presented for this study (endpoint control group measurements not included due to small sample size). Fallingborg et al. [72] and Monnard et al. [69] did not report standard deviations or any other measures of spread, hence no error bars. Haase et al. [67] capsule 1 data not included in plot as this data was reanalysed and pooled in the Nandhra et al. [5] study. ColorBrewer colour scheme used for plots www.ColorBrewer.org (accessed on 25 May 2023) [82].

Seventeen (77%) of the twenty-two included studies reported GET (Table 3), providing a total of twenty-three GET values ranging between 0.4 and 15.3 h (Figure 2a). The sample size for these studies ranged between 20 and 580 subjects. Six studies [63,65,70,74,75,78] measured GET using imaging systems (GET ranging between 0.4 and 0.8 h), four studies [5,30,67,68] used magnetic/electromagnetic systems (GET ranging between 1.0 and 7.1 h), seven studies [6,55,61,72,73,77,79] used pH-sensing systems (GET estimates ranging between 0.8 and 15.3 h) and one study [61] used a gas sensing system (GET estimates ranging between 2.4 and 2.5 h).

**Figure 3:**
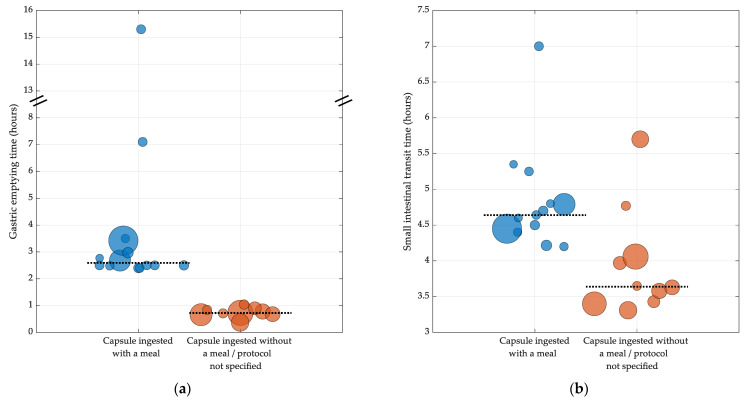
Influence of ingestion meal protocol on (**a**) gastric emptying time and (**b**) small intestinal transit time. Blue bubbles represent transit time values obtained from capsules ingested with a meal. Orange bubbles represent transit time values for capsules ingested without a meal or where the ingestion meal protocol was not specified. Dotted line represents the median of transit time values. Each point is weighted by the sample size. Note: It was not possible to illustrate the impact of protocol.

In eight studies [5,6,54,61,67,68,77,79], capsules were ingested with a meal, with a total calorific content ranging between 255 and 964 kcal. These studies reported longer GET values (ranging between 2.4 and 15.3 h) than those where capsules were either ingested with water or where the ingestion protocol was not specified (reported GET values of ≤1 h) (Figure 3a). Of particular note is the result from Koziolek et al. [54], which reported the longest GET value of 15.3 h. The subjects in this study ingested the capsule with a high calorie meal (964 kcal) and fasted for a shorter duration following capsule ingestion (4.5 h) than the subjects in other studies who ingested capsules with a lower calorie meal (ranging between 255 and 602 kcal) and fasted for a longer duration (6 h); these studies reported GET values ranging between 2.5 and 7.1 h. In three of the studies [30,55,74], the subjects ingested capsules with water only. These studies reported GET values between 0.4 and 1.0 h (Figure 3a). Six studies [63,65,70,72,75,78] did not specify an ingestion meal protocol (all but one [72] of these studies used intraluminal imaging systems). These studies reported GET values between 0.7 and 0.9 h (Figure 3a).

In 12 studies [5,6,30,55,61,65,67,72,73,74,77,79], the subjects ingested the capsule in the morning. These studies reported GET values ranging between 0.4 and 15.3 h. The study that utilised multiple capsules [67] required its subjects to ingest the second capsule in the evening (median GET value: 7.1 h) and the third the next day in the morning (median GET value: 3.5 h). Five studies [63,68,70,75,81] did not specify the capsule ingestion time; these studies reported GET values ranging between 0.7 and 2.5 h. Twelve studies specified the fasting duration following capsule ingestion. Fallingborg et al. [72] required subjects to fast until the capsule was seen to exit the stomach, as indicated by a pH rise. This study reported a GET value of 0.9 h. Two studies [65,73] required the subjects to ingest a liquid meal or water 45–60 min following capsule ingestion. These studies reported GET values of 0.7 and 15.3 h, respectively (note that the subjects in the Koziolek et al. [73] study ingested of a solid meal 4.5 h after capsule ingestion). Two studies [55,74] required the subjects to fast for 4 h following capsule ingestion, resulting in GET values ranging between 0.4 to 0.8 h. The remaining seven studies [5,6,61,67,68,77,79] required the subjects to ingest the capsule with a meal and then fast for 6 h. These studies reported GET values between 2.4 and 7.1 h. Five studies [30,63,70,75,78] did not specify a fasting protocol following capsule ingestion. These studies reported GET values between 0.7 and 1.0 h.

#### 3.5.2. Small Intestinal Transit Time

SITT estimates were reported by seventeen (77%) of the included studies (Table 3), providing a total of twenty-three SITT values ranging between 3.3 and 7 h (see Figure 2b). The sample sizes of the studies ranged between 20 and 215 subjects. Eight studies [64,65,66,70,74,75,76,78] measured SITT using imaging systems (SITT ranged between 3.3 and 4.1 h), three studies [5,67,68] used electromagnetic systems (SITT ranged between 4.6 and 7 h), six studies [6,55,61,71,77,79] used pH-sensing systems (SITT ranged between 4.2 and 5.7 h) and one study [61] used the gas sensing capsule, reporting SITT values between 4.2 and 4.8 h. In seven [5,6,61,67,68,77,79] of the seventeen studies that reported SITT, the subjects ingested the capsule with a meal. These studies reported SITT values between 4.2 and 7 h (Figure 3b). Three studies [55,71,74] specified that the subjects ingested the capsule with water. These studies reported SITT values between 3.3 and 5.7 h (Figure 3b). The remaining seven studies [64,65,66,70,75,76,78] did not specify an ingestion protocol. These studies used imaging systems and reported SITT values between 3.4 and 4.1 h (Figure 3b).

#### 3.5.3. Colonic Transit Time

Of the twenty-two included studies, only nine (41%) reported CTT estimates (Table 3), providing a total of fifteen CTT values, ranging between 15.9 and 28.9 h (see Figure 2c). The sample size of the studies ranged between 20 and 215 subjects. Three studies [5,67,68] measured CTT using electromagnetic systems (CTT ranging between 15.9 and 25.2 h), five studies [6,55,61,77,80] used pH-sensing systems (CTT estimates ranging between 19.0 and 28.5 h), one study [76] used an imaging system (reported CTT value: 28.9 h) and one study [61] used the gas sensing capsule (CTT estimates ranging between 19.2 and 20.2 h). Seven studies [5,6,55,61,67,77,79] required the subjects to ingest the capsules in the morning, reporting transit times between 17.6 h and 28.5 h. The subjects swallowed the second capsule in the study by Haase et al. [67] in the evening, resulting in a median CTT value of 25.2 h (the median corresponding CTT for capsules swallowed in the morning was 18.2 h and those swallowed the next day in the morning was 17.6 h). Two studies [68,76] did not report the capsule ingestion time. These studies reported CTT values between 15.9 h and 28.8 h.

#### 3.5.4. Whole-Gut Transit Time

Eleven (50%) of the twenty-two included studies (see Table 2) reported WGTT, providing a total of seventeen values, ranging between 23.0 h and 37.4 h (six results reported as means and eleven as medians)—see Figure 2d. The study sample sizes ranged between 20 and 215 subjects. Six studies [6,55,61,71,77,79] used pH-sensing systems (WGTT ranged between 23.3 h and 36.5 h), three studies [5,67,68] used electromagnetic systems (WGTT ranged between 23.0 h and 37.4 h), one study [76] used an imaging system (WGTT estimate—38.4 h), one study [69] used a temperature sensing system (mean WGTT: 31 h) and one study [61] used the gas sensing capsule (WGTT estimates ranging between 25.8 and 26.9 h). With regards to the capsule ingestion time, eight studies [5,6,55,61,67,71,77,79] required the subjects to ingest the capsules in the morning. For these studies, the WGTT ranged between 23.3 h and 36.5 h. In one study [69], the subjects ingested the capsule in the afternoon, reporting a mean WGTT of 31.0 h. The second capsule in the study by Haase et al. [67] was ingested in the evening, which yielded a median WGTT of 37.4 h (the corresponding WGTT for capsules swallowed in the morning on days 1 and 2 were 27.6 h and 25.0 h). Two studies [68,76] did not specify the capsule ingestion time; these studies reported WGTT ranging between 23.0 and 33.9 h.

## 4. Discussion

The primary objectives of this review were to: firstly, provide an overview of the currently available IC systems, and secondly, perform a systematic review of studies utilising these systems to report GI transit times in healthy volunteers. Two systematic reviews were identified on a similar topic as part of the search [83,84]; however, these studies differ from our systematic review as they did not report GI transit times [83], or in the case of the review by Abuhelwa et al. [84], the literature search was not exclusive to IC systems, i.e., GI transit times were predominantly reported from studies that utilised non-disintegrating dosage forms/radiolabelled tablets tracked using scintigraphy, and a few studies using pH-sensing capsules and magnetically marked pellets. This systematic review is therefore the first of its kind to present and report GI transit times in healthy adults from IC systems.

### 4.1. Summary of Evidence

#### 4.1.1. Gastric Emptying Time

The majority of included studies reported GET values, albeit over a large range. This appears to be due to variations in the protocol, which have an impact on GET, especially if capsules are ingested with a meal. Non-digestible solids have been shown to empty from the stomach in its fasting state, when powerful antral phase III MMC contractions occur, which expel undigested content from the stomach [85,86]. For this to happen, the stomach must switch from the ‘fed’ to ‘fasting’ state, and this occurs once a meal has been emptied from the stomach. How quickly this happens depends on several factors, such as the total caloric content of the meal, where higher caloric meals result in a prolonged GET [84,85]. In the fasted state, the MMC cycle recurs every 130 min [87]. However, a solid digestible meal can disrupt the MMC for up to 4 h [88], depending on the caloric content of the meal; hence, capsules ingested with liquids empty from the stomach quicker than those ingested with a solid meal. Fasting duration also has an impact on how quickly a non-digestible solid empties from the stomach, where shorter intervals between meals result in a prolonged fed state, delaying the onset of the MMC cycle [85]. In the study by Koziolek et al. [73], the subjects ingested a high-calorie ingestion meal and fasted for less than 6 h following capsule ingestion. This may explain the exceptionally long GET values reported by this study, as these conditions are likely to keep the stomach in the “fed” state for a prolonged period of time, thereby delaying the onset of the phase III MMC contractions. The capsule ingestion time also affects GET, as reported by Haase et al. [67] where the ingestion of a capsule in the evening delays GET due to reduced motility during sleep [89].

Variations in the study protocol make it difficult to provide concise reference ranges for GET as measured using IC systems. However, it is not possible for all capsule studies to adopt the same protocol for the purposes of measuring GI transit times as, for some systems, such as the imaging capsules, transit times are only a secondary measure; therefore, capsules need to be ingested without a meal to ensure clear images of the gut mucosa are acquired. Nonetheless, the results for GET from the included studies can be grouped based on two similar protocols if the results from Koziolek et al. [73] and the evening capsule data from Haase et al. [67] are excluded due to extreme variations in the protocol: (1) studies that required subjects to ingest the capsule with a standard-calorie meal [5,6,61,67,68,77,79], which reported GET values between 2.4 and 3.5 h; (2) studies where subjects ingested the capsule with a liquid [55,63,65,70,72,74,75,78]. This includes the studies that did not specify whether the capsule was ingested with a meal as the majority of these studies utilised imaging capsules; therefore, it is safe to assume that the capsules were ingested with water. These studies reported GET values ranging between 0.4 and 1.0 h.

#### 4.1.2. Small Intestinal Transit Time

SITT measurements were readily available from most of the included studies, with the reported values falling over a relatively large range. Interestingly, the studies that used imaging capsules reported shorter SITT values (<4 h) than those that used electromagnetic or pH sensing systems, which reported values > 4 h. This may be due to variations in the study protocol as the majority of studies that used imaging capsules did not specify whether the capsule was ingested with a meal. It is highly likely that these capsules were ingested without a meal for the purposes of obtaining clearer images. Therefore, similar to GET, the shorter SITT values obtained from imaging capsules may perhaps be due to the activity of the small intestine’s MMC, which may speed up the progress of non-digestible solids in the absence of any food or liquids that would have otherwise disrupted the activity in the small intestine [90]. However, the meta-analysis by Abuhelwa et. al. [84] reported no effect of food on SITT. Additionally, two studies that used pH sensing systems [55,71] specified that the capsule was ingested with water and reported SITT values > 4 h. As with most capsule-based systems, the question of whether an indigestible solid can provide an accurate physiological measure of the transit of fluid-like ingested content is critical when it comes to interpreting the results. This is most apparent with GET, where a non-digestible solid empties after the digestible meal and may therefore result in longer estimates for transit times. Additionally, factors such as the dimensions of the capsules, the protocols and the method of analysis may well influence the resulting estimates. This warrants further research into the effects of a meal on the transit of non-digestible solids in the small intestine. Similar to GET, the capsule ingestion time also appears to have an effect on SITT, whereby capsules ingested in the evening measure longer SITT values due to reduced gut motility [89]. It is not clear whether fasting has an impact on SITT as the ranges appear to overlap. Fadda et al. [91] reported no differences between the fasted and fed states on the SITT; however, these results were obtained from scintigraphy studies.

#### 4.1.3. Colonic Transit Time

Normal reference values for CTT have been shown to fall over a large range of ≤70 h in mixed populations [92]. Additionally, studies using different IC systems [5,6], as well as other methods, such as the “blue poo” method [93], have shown that CTTs and WGTTs occur at peak times, separated by 24 h, reflecting normal human bowel habits [94]. Although colonic scintigraphy and radio opaque marker studies (ROM) are the standard methods for measuring CTT [92], IC systems have the ability to measure whole- and segmental-CTT over prolonged periods of time [5,6,95]. However, only a few eligible studies reported CTTs. This is not surprising as almost half of the included studies used the PillCam SB imaging capsule (Medtronic, Minneapolis, MN, USA), which is not suitable for use in the colon due to a short battery life. For this, the PillCam COLON was developed; however, studies [44,96] using this capsule performed extensive bowel preparation and were carried out in patients, and therefore did not meet the inclusion criteria. Surprisingly, the OMOM imaging capsule used by Jianqin et al. [76] was used to estimate CTT and even WGTT, having a battery life of just 12 h, like the PillCam SB. Although not indicated in the study methods, it is likely that the capsule ingestion and expulsion times, along with the estimates of GET (which were not reported) and SITT were used to determine CTT and WGTT. The capsules therefore identified as being the most suitable for assessing colonic transit are the pH sensing and electromagnetic capsules due to a longer battery life.

#### 4.1.4. Whole-Gut Transit Time

ROM studies are the established methods for the assessment of WGTT [4], with reported mean values of ~50 h [97] in healthy adults. However, unlike imaging methods such as scintigraphy and ROM studies, which can only estimate WGTT from snapshot measurements, IC systems can continuously measure WGTT from the point of capsule ingestion to expulsion, thereby providing a more comprehensive estimate. The results of this systematic review show that the estimates from IC systems are consistent across all systems. However, the most appropriate IC systems for measuring WGTT appear to be the electromagnetic, pH and temperature sensing systems as the estimates are derived from their sensor measurements. Imaging capsule systems are not designed for long-term measurements like WGTT due to the short battery life; however, the WGTT can be determined using the time that the capsule was ingested and expelled.

#### 4.1.5. Limitations

There are several limitations to this systematic review. Firstly, it was not possible to perform a quantitative meta-analysis of GI transit times due to the heterogeneity of the included studies and associated methodologies. Therefore, we were not able to determine effect sizes or quantitatively analyse the influence of factors such as the ingestion protocol on GI transit times. The study quality was also a limiting factor as only five of the twenty-two included studies were of a high quality. The limitation on the sample size of the eligible studies to twenty subjects was chosen for pragmatic reasons; however, six of the included studies [61,67,68,73,75,77] reported GI transit times for <20 subjects, but were still included in this review as they met the inclusion criteria for intention-to-investigate transit times in 20 subjects. True estimates of transit times would require larger sample sizes. The influence of gender on GI transit times is well-known [5], and performing a quantitative analysis by gender may have been insightful. However, this was not undertaken due to the heterogeneity of the study protocols and the inability to clearly extract the results of males and females from most of the studies.

Finally, only two [61,67] of the twenty-two included studies assessed the reproducibility of the measured gut transit times, as illustrated in Figure 2. The subjects in the study by Haase et al. [67] ingested three 3D-Transit capsules over a period of two consecutive days. Day-to-day and intrasubject variations were noted to be high, even for capsules that were ingested under a similar protocol (coefficient of variation (CV) ranging between 20% and 45% for all transit times) [67]. Thwaites et al. [61] determined intrasubject variability through the tandem-ingestion of two gas sensing capsules and observed CVs between 11–35% for all transit times, with the colon displaying the most variability. Both studies concluded that the variances relate to physiological, rather than methodological differences.

## 5. Conclusions

The ambulatory monitoring of GI motility over extended periods of time outside clinical or laboratory environments has been made possible by IC systems, thereby expanding our knowledge of normal and pathological GI function. This systematic review has shown that there are a variety of IC systems available for the assessment of GI motility, and despite the differences in the sensor technologies employed, all of the systems appear to be capable of measuring GI transit times within a minimally invasive and radiation-free setting. However, a lack of standardisation of the protocols, even for the same type of IC system, makes it difficult to compare and combine the results. Nevertheless, reference ranges for GI transit times in healthy volunteers as determined using IC systems have been produced (Table 4), which show that the measurements are relatively consistent across the different IC systems used in the included studies.

## Figures and Tables

**Table 1:** Inclusion and exclusion criteria in terms of participants, interventions, comparisons, outcomes and study design (PICOS) for the systematic review.

	Inclusion Criteria	Exclusion Criteria
Participants	Healthy adults (>18 years)	Patients Pediatric populations (<18 years) Animals
Interventions	Use of free-falling ingestible capsule systems to measure gastrointestinal transit times as primary or secondary outcome measures. This includes commercially available systems and those that are still in the research domain	Use of: Radio-opaque marker studies Radiolabeled meals or labelled drug-dosage forms tracked using scintigraphy techniques or other imaging techniques e.g., MRI Any other systems or techniques that did not utilise IC system were also excluded. Tethered capsules Magnetically/robotically controlled capsules following ingestion e.g., MACE Medicine adherence capsules or drugs with digital ingestion tracking system
		Any studies that performed extensive bowel cleansing prior to capsule ingestion or involved an intervention or treatment that could affect GI transit times
Comparisons	Not applicable	Not applicable
Outcomes	Gastric emptying time (GET) Small intestinal transit time (SITT) Colonic transit time (CTT) Whole-gut transit time (WGTT)	Not applicable
Study designs	Randomised controlled trials, observational cohort and cross-sectional studies, case-control studies, case series, before-after (pre-post) studies with no control group The selection of studies was not solely restricted to healthy volunteer studies, i.e., any clinical studies involving patients and including a healthy volunteer group as a comparator were also included Intention to investigate transit times as either primary or secondary measures in at least 20 subjects	Review articles Abstracts Case reports Opinions Letters Comments Editorials

**Table 2:** Capsule ingestible systems capable of measuring gastrointestinal transit times.

Device	Year Introduced	Sensing Capabilities/Indications for Use	System Components	System Component Dimensions	Measurement Range and Accuracy	Battery Life	Transit Time Measurement Capabilities				Measurement Validation
							GET	SITT	CTT	WGTT	
Heidelberg Capsule (Heidelberg Medical, Germany) [8,9]	~1960s	Sensor: gut pH; Commercially available.	Ingestible pH capsule; Transceiver; Interface Module; pH capsule locator; Dedicated software.	Capsule Ø 8 mm, length 21 mm	pH measurement range: 1–8 pH units pH accuracy: ±0.5 pH units	Transceiver: 12–14 h	✓				Acid values obtained from the capsule were compared against tube aspiration and showed acceptable correlation [10]. No comparative transit time studies.
Radiotelemetry Capsule (RTC) (Remote Control Systems Ltd., Consett, UK) * [11,12]	1981	Sensor: gut pH; Research use only.	Ingestible pH capsule; Portable solid-state receiver.	Capsule Ø 7.6 mm, length 26 mm Solid state receiver: 400 g	pH measurement range: 1–10 pH units pH accuracy: ±0.2 pH units	24 h (Fs = 6 s) or up to 48 h (Fs = 12 s)		✓		✓	No comparative transit time studies.
CorTemp^®^ (HQ, Inc., Palmetto, FL, USA) * [13,14]	~1988	Sensor: core body temperature; Commercially available.	Ingestible capsule; Data recorder; Dedicated software (CorTrack^®^ II).	Capsule Ø 10.9 mm, length 22.4 mm, weight 2.8 g	Operating range: 30 °C to 45 °C Accuracy: 0.27 °C	7–10 days				✓	Good reliability when compared against oesophageal and rectal temperatures, however capsule mobility results in measurement variability [13]. Water bath validation shows excellent validity and test-retest reliability, after removal of outlier [14]. No comparative transit time studies.
Magnetic Marker Monitoring System (Department of Biopharmaceutics and Pharmaceutical Technology, University of Greifswald, Greifswald, Germany) [15,16]	1994	Sensor: magnetic; GI localisation for real-time tracking of dosage forms; Research use only.	Ingestible magnetised tablets containing drug and ferromagnetic black iron oxide; Biomagnetic measurement device containing SQUIDs.	Tablet Ø 11 mm Biomagnetic measurement device coverage range: Ø 230 mm	High spatial and temporal resolution in the range of 1 mm	N/A Mains powered system	✓	✓			Experimental set-ups using test objects of known dimensions demonstrates high spatial and temporal resolution [16]. No comparative transit time studies.
PillCam™ SB (Medtronic Inc., Minneapolis, MN, USA) * [17,18,19]	2000	Sensor: video camera; Indicated for obscure GI bleeding and the diagnosis and investigation of Crohn’s disease [17]; Commercially available.	Ingestible video capsule; Sensor belt and sensor array; Data recorder; Dedicated software (PillCam^TM^ software v9)	Capsule Ø 11.4 mm, length 26.2 mm, weight 3 g	Minimum size of detection—0.07 mm Image resolution—340 × 340 Frame rate: 2–6 fps Field of view: 156°	8–12 h	✓	✓			PillCam yields shorter GET and SITT than WMC [19].
Motility Tracking System (Motilis Medica SA, Lausanne, Switzerland) [20,21,22]	2002	Sensor: magnetic. Regional GI transit times and motility patterns e.g., regional contraction frequencies, velocities, segment lengths and direction of movement GI localisation for real-time tracking; Research use only.	Ingestible magnetic capsule (permanent magnet); 4 × 4 matrix of sensors; Dedicated software (MTS_Record)	Capsule Ø 6 mm, length 15 mm	Position accuracy: ±5%	Unknown	✓	✓			Good agreement seen in GET and SITT values obtained from the MTS capsule and PillCam [22].
WMC (SmartPill™) (Medtronic Inc., Minneapolis, MN, USA) * [23,24]	2003	Sensor: gut pH, temperature and pressure. Indicated for the evaluation of GI motility disorders e.g., suspected delayed gastric emptying and differentiation between normal and slow transit constipation [25]; Commercially available.	Ingestible capsule; Data receiver; Dedicated software (MotiliGI^TM^ Software)	Capsule Ø 26.8 mm, length 11.7 mm, weight 4.5 g Receiver: approx. 150 mm × 100 mm × 38 mm	Pressure range: −0–350 mmHg Pressure accuracy: ±5 mmHg below 100 mmHg Temperature range: 25–49 °C Temperature accuracy: ±0.5 °C pH measurement range: 0.05–9.0 pH units pH accuracy: ±0.5 pH units	Capsule and Data receiver, >5 days	✓	✓	✓	✓	Several transit studies have shown good agreement between WMC and ROMs or scintigraphy [26,27].
3D-MAGMA (Matesy GmbH, Jena, Germany) * [28,29]	2003	Sensor: magnetic; Real-time tracking of magnetic markers for the measurement of gut contraction frequencies and power, transit times marker progression paths and velocities; Commercially available.	Ingestible permanent magnetic capsule; Sensor system containing 27 magnetic field sensors. Dedicated software	Capsule Ø 6 mm, length 16 mm, density ~<1.5 g/cm^3^	Realtime position tracking accuracy: 3 mm	N/A Mains-powered system	✓				Strong linear correlation between 3D-MAGMA and Electrogastrography for the measurement of gastric slow waves [30]. No comparative transit time studies found
OMOM^®^ (Jinshan Science and Technology Company, Chongqing, China) * [31,32]	2004	Sensor: video camera; For small bowel evaluation.	Ingestible video capsule; Portable image recorder. Dedicated software (SmartScan, SmartView, SmartFinding, Vue Smart)	Capsule Ø 11 mm, length 25.4 mm, weight: 3 g	Depth of field: 0–50 mm Minimum size of detection—0.1 mm Image resolution—512 × 512 Frame rate: 2–10 fps Field of view: 172°	12 h		✓	✓	✓	Diagnostic yield, functionality and SITT of OMOM compared against PillCam SB3. No statistically significant difference found between the two systems [33].
VitalSense^®^ (Philips Respironics, OR, USA) [14,34]	2004	Sensor: core body temperature; Commercially available.	Ingestible capsule; Data recorder; Dedicated software (Equivital Manager v1.2.39.4600).	Capsule Ø 8.7 mm, length 23 mm, weight: 1.5 g	Operating range: −10 °C to 60 °C Accuracy: 0.17 °C	10 days				✓	No significant differences between capsule and rectal measure temperatures [34]. Good validity and test-retest reliability in water bath, after removal of outliers [14]. No comparative transit time studies.
EndoCapsule (Olympus Inc., Tokyo, Japan) [35]	2005	Sensor: video camera; visualisation of small intestinal mucosa; Commercially available.	Ingestible capsule; Recorder; Battery pack; Antenna Unit; Capsule activator; Recorder; antenna holder and cradle; Dedicated software (ENDOCAPSULE SOFTWARE 10)	Capsule Ø 11 mm, length 26 mm, weight: 3.3 g Recorder: 87 mm × 154 mm × 33 mm, weight: 320 g Battery pack: 70 mm × 10 mm × 55 mm, weight: 70 g Antenna: 87 mm × 51 mm × 15 mm, weight: 150 g	Field of view: 160° Depth of field: 0–20 mm Frames per second: 2	Capsule: 12 h Recorder: 12 h	✓	✓			No significant difference in mean SITT between EndoCapsule and PillCam SB in patients with OGIB [36]. Similarly, no statistically significant difference in GET and SITT between EndoCapsule and MiroCam as measured in patients referred for VCE [37].
Experimental system Institute of Precision Engineering and Intelligent Microsystem, Shanghai Jiaotong University, Shanghai, China [38]	2005	Sensor: gut pH, temperature and pressure; Research use only.	2 indigestible biotelemetry capsules: PT (pressure and temperature sensing) pH sensing Data recorder; Ultrasonic electrode waistcoat; Dedicated software.	Capsule Ø 10 mm, length 21.1 mm, weight: 2.9 g pH capsule: Ø 10 mm, length 24 mm, weight: 5.2 g	Pressure range: −60–200 mmHg Pressure accuracy: 1% Temperature range: 34–42 °C Temperature accuracy: ±0.2 °C pH measurement range: 1–13 pH units pH accuracy: ±0.2 pH units	Unknown				✓	Laboratory tests performed by a test house, measuring against gauge data, verifying feasibility and functionality [38]. No comparative transit time studies.
CapsoCam^®^ (CapsoVision, Cupertino, CA, USA) [39]	2006	Sensor: video camera; Provides a 360° panoramic view of the small bowel mucosa; Commercially available.	Ingestible capsule with on-board data storage capabilities, avoiding the need for external recording equipment. Dedicated software (CapsoVision)	CapsoCam Plus capsule: Ø 11 mm, length 31 mm Weight: 4 g	Image resolution 221,184 Max frame rate: 20 fps Field of view: 360° Depth of view: 0–18 mm No. of cameras: 4	15 h	✓	✓			Several patient studies performed comparing diagnostic findings of CapsoCam against PillCam SB. No statistically significant differences found in GET and SITT between the two systems [40,41,42].
PillCam™ Colon (Medtronic Inc., Minneapolis, MN, USA) [43]	2006	Sensor: video camera; colon capsule endoscopy for polyp detection, diagnosing inflammatory bowel disease and colorectal cancer screening; Commercially available.	Ingestible video capsule; Sensor belt and sensor array; Data recorder; Dedicated software (PillCam^TM^ software v9)	Capsule Ø 11.6 mm, length 32.3 mm, weight: 2.9 g	Field of View: 172°; Minimum detectable object: at least 0.1 mm; Frame rate: 4–35 fps	10 h			✓	✓	Validated against colonoscopy for detection of colorectal polyps/other diseases [44,45,46]. No comparative transit time studies found.
MiroCam^®^ (IntroMedic Co., Seoul, Republic of Korea) [18,47]	2009	Sensor: video camera; exploration of entire small bowel; Commercially available.	Ingestible video capsule; Sensor pads (images transmitted via Human Body Communication); Receiver; Dedicated software (MiroView^TM^ software)	Capsule Ø 10.8 mm, length 24 mm, weight: 3.3 g	Image resolution—320 × 320 Frames per second: 2 Field of view: 150°	9–11 h	✓	✓			Several studies compared the diagnostic yield of the MiroCam against other capsule endoscopy systems e.g., PillCam [48] or EndoCapsule [37] but no comparison of transit times.
Bravo™ pH capsule (Medtronic Inc., Minneapolis, MN, USA) [49]	2011	Sensor: oesophageal pH; Indicated for gastro-oesophageal reflux monitoring; Commercially available.	Ingestible capsule; Data recorder; Dedicated software (Bravo^TM^ Reflux Recorder)	Capsule 5 mm × 6 mm × 25 mm, weight: 1.5 g	pH measurement range: 0.5–9.0 pH units	Up to 96 h	✓	✓			Transit times obtained from capsule compared against those obtained from radiolabelled tablets. Some differences possibly due to size differences between capsule and tables [49].
3D-Transit (Motilis Medica SA, Lausanne, Switzerland) * [50,51]	2012	Sensor: electromagnetic; Regional and segmental GI transit times and motility patterns e.g., regional contraction frequencies, velocities, segment lengths and direction of movement GI localisation for real-time tracking. Research use only.	Ingestible electromagnetic capsule; Detector plate and power supply; Respiration measurement belt; Dedicated software (MTS2 software)	Capsule Ø 8.3 mm, length 23 mm, weight: 1.8 g Detector plate: 160 mm × 160 mm × 11 mm; weight: 145 g	Detector range: 4–40 cm (not indicated for abdominal diameter > 140 cm) Absolute position inaccuracy: 10% of the distance between the capsule and the detector at the maximum.	Capsule: 60 h (at 10 Hz) and 120 h (at 5 Hz)	✓	✓	✓	✓	No direct transit time comparison studies against other methods however, good inter and intra-rater reliability of measurements seen [5,52].
IntelliCap^®^ (Medimetrics, Eindhoven, The Netherlands) * [53,54]	2013	Sensor: gut pH and temperature sensing; For electronic drug delivery and monitoring; Commercially available.	Ingestible capsule; Start-up unit to program and activate capsule; Portable recording unit that transmits data to a PC; Dedicated software	Capsule Ø 11 mm, length 27 mm	Relative pH accuracy: ±0.3 pH units Relative temperature accuracy: ±0.1 °C	Capsule battery lasts at least 48 h	✓	✓	✓	✓	Capsule localisation compared to scintigraphy and shown to correlate well [55]. No comparative transit time studies found.
C-scan^®^ system (Check-Cap Inc., Isfiya, Israel) [56,57]	2014	Sensor: ultra-low dose X-ray source (Tungsten 181 Radioisotope); temperature, pressure and radio frequency signalling; For polyp detection; Commercially available.	Ingestible Capsule (C-Scan^®^ Cap); Recorder (C-Scan ^®^Track); Dedicated workstation; Dedicated software (C-Scan^®^ View).	Capsule Ø 11.6 mm, length 34 mm	Capsule position and orientation accuracy in colon: ±1 cm	Capsule: battery lasts 100 h				✓	76% sensitivity and 82% specificity for the detection of precancerous polyps when compared to fecal immunochemical test [58].
MyTemp (MyTemp, Nijmegen, The Netherlands) [14]	~2016	Sensor: core body temperature; Research use only.	Ingestible capsule; Copper-wired waistband; Dedicated software (myTemp manager v01.08).	Capsule Ø 8 mm, length 20 mm, weight: 1.3 g	Operating range: 30 °C to 45 °C Accuracy: ±0.001 °C	Infinite (no battery—self-induction)				✓	Water bath validation shows excellent validity and test-retest reliability, after removal of outliers [14]. No comparative transit time studies.
e-Celsius^®^ (BodyCap, Caen, France) [14]	CE-marked version introduced in 2017	Sensor: core body temperature; Commercially available.	Ingestible capsule; External recorder; Dedicated software (e-Performance manager v01.01.00.0C).	Capsule Ø 8.9 mm, length 17.7 mm, weight: 1.7 g	Operating range: −0 °C to 50 °C Accuracy: ±0.23 °C	20 days				✓	Water bath validation shows excellent validity and test-retest reliability, after removal of outliers [14]. No comparative transit time studies.
Gas sensing capsule (Atmo Biosciences, Box Hill, VIC, Australia) * [59,60,61]	2018	Sensors: temperature, relative humidity, hydrogen and carbon dioxide concentration, along with concentrations of total relative volatile organic compounds, capsule orientation and changes in the physical electromagnetic properties of the capsule’s environment [61]. Measures gas concentrations in aerobic and anaerobic conditions within the gut; Research use only.	Ingestible gas sensing capsule; Handheld receiver; Mobile phone software application.	Capsule Ø 11 mm, length 28 mm	Gas sensing accuracy of earlier versions of the capsule: Hydrogen and oxygen better than 0.2% and Carbon dioxide—1% [59]. Sensor range and accuracy of latest version not currently in publication	Capsule: 4 days Temperature sensor and transmission circuitry~30 days	✓	✓	✓	✓	Anatomical landmarks as determined using the gas sensing capsule was validated by concurrent (tandem) ingestion of the WMC—good agreement in transit time measurements [61].
MoPill^TM^ (Texas Tech University Health Sciences Center, Lubbock, TX, USA) [62]	2021	Sensor: radio frequency (RF) signalling RF position system; Regional and segmental GI transit times GI localisation for real-time tracking; Research use only.	Ingestible capsule; 4 adhesive sensors—2 for abdomen and 2 for back; Recorder; Dedicated software.	Capsule Ø 12 mm, length 20 mm Adhesive sensors: 60 mm × 55 mm	Localisation accuracy range: 9–94 mm	Unknown	✓	✓	✓	✓	Capsule location validated using X-ray imaging [62].

* IC systems used in studies meeting the inclusion criteria of the systematic review. N/A: Not applicable.

**Table 4:** Reference ranges for GI transit times as measured using IC systems.

Parameter		Minimum Reported Value (Hours)	Maximum Reported Value (Hours)
Gastric emptying time	Capsule ingested without a meal	0.4	1.0
	Capsule ingested with a meal *	2.4	3.5
Small intestinal transit time		3.3	5.7
Colonic transit time		15.9	28.9
Whole gut transit time		23.0	37.4

* Excluding results from Haase et al. [67] (capsule 2 data) and Koziolek et al. [73] due to extreme variations in protocol.

## Data Availability

Data can be made available on written request.

## Supplementary Materials

The following supporting information can be downloaded at: https://www.mdpi.com/article/10.3390/jcm12165272/s1: Section S1: Search Strategy—Table S1: Search strategy queries; Section S2: Results of Literature Review; Section S3: List of studies utilising data from the same healthy volunteer cohorts; Section S4: Results of Bias Assessment—Table S2: Scores for before-after studies; Table S3: Scores for case-series studies; Table S4: Scores for cohort and cross-sectional studies; Table S5: Scores for randomised controlled trial studies; Table S6: Scores for case-control studies; Figure S1: Bias assessment scores.
