# Supplementary material for: Gastrointestinal Transit Times in Health as Determined Using Ingestible Capsule Systems: A Systematic Review
Source: J Clin Med. 2023 Aug 13;12(16):5272. doi: 10.3390/jcm12165272 (PMC10455695; doi:10.3390/jcm12165272)

Journal of Clinical Medicine

Systematic Review

Gastrointestinal Transit Times in Health as Determined Using Ingestible Capsule Systems

Gursharan Kaur Nandhra [1,2,*], Phakanant Chaichanavichkij [1], Malcolm Birch [1,2] and S. Mark Scott [1]

1 National Bowel Research Centre and GI Physiology Unit, Blizard Institute, Centre for Neuroscience, Surgery & Trauma, Queen Mary University of London, London E1 4NS, UK; g.k.nandhra@qmul.ac.uk (G.K.N.); p.chaichanavichkij@qmul.ac.uk (P.C.); m.j.birch@qmul.ac.uk (M.B.); m.scott@qmul.ac.uk (S.M.S.)
2 Clinical Physics, Barts Health NHS Trust, The Royal London Hospital, London E1 2BL, UK
* Correspondence: g.k.nandhra@qmul.ac.uk; Tel.: +44-(0)20-359-46596

Supplementary Material

Section S1: Search strategy

Table S1: Search strategy queries

Query No.	Search strategies
1.	((gastrointestin*) OR (gut) OR "gastric empty*" OR "small intestin*" OR "small bowel" OR colon* OR "large bowel") AND (motility) AND (capsule* OR pill*)
2.	("capsule endoscopy" OR pH AND (capsule OR pill) OR ((temperature OR "core body temperature") AND (ingestible OR capsule* OR pill*)) OR ((magnet* OR electromagnet*) AND (capsule OR pill) AND tracking)) AND (gastrointest* OR gut) AND transit
3.	(PillCam OR EndoCapsule OR OMOM OR MiroCam OR Capsocam OR ("Wireless Motility Capsule" OR WMC OR SmartPill) OR IntelliCap OR CorTemp OR VitalSense OR e-Celsius OR (Gas sensing AND (capsule OR pill)) OR ("Heidelberg capsule") OR ("Radiotelemetry Capsule") OR ("Bravo pH Capsule") OR "magnetic marker monitoring" OR ("Motility Tracking System") OR ("3D-Transit")) AND ((gastroint* OR gut) OR "gastric empty*" OR "small intestin*" OR "small bowel" OR colon* OR "large bowel")

Section S2: Results of Literature Review

S2.1. Intraluminal imaging systems

Intraluminal imaging systems were the first to be clinically applied since the Endoradiosonde. The PillCam (formerly Given Imaging, Yokneam, Israel; now Medtronic, Minneapolis, MN, USA), also known as wireless capsule endoscopy (WCE) was the first commercially-available IC system [1, 2], developed as a non-invasive, pain-free alternative to endoscopy, aiming to target inaccessible regions of the gut such as the small intestine. It consists of a single-use capsule containing high-resolution cameras, microcontrollers, antennas, light emitting diodes (LEDs) and batteries [3, 4]. The system acquires approximately 50,000 images per recording, transmitted using radiotelemetry to eight aerials attached to the body, which also estimate the position of the capsule in the body using the signal strength received by each aerial. The received images are stored on a small portable recorder attached to a belt and worn around the abdomen. Bespoke software is used to visualise the video sequences, which can take between 45 minutes to 2 hours to analyse, depending on the experience of the examiner. Although the PillCam is the most widely used WCE system, several different variations are now available from other manufacturers [4]. These include the EndoCapsule (Olympus Inc., Tokyo, Japan)[5], OMOM Capsule (Jinshan Science and Technology Company, Chongqing, China)[6], MiroCam (Intromedic Co., Seoul, South Korea), which uses human body communication rather than radio frequency communication to transmit signals [7], and CapsoCam (CapsoVision, Cupertino, CA, USA) [8]. As described, the primary outcome of intraluminal imaging capsule systems are the images, which are used to visualise and assess gut morphology. Gut transit times are a secondary outcome, which can be estimated from certain landmarks as identified in the images e.g. gastric emptying (GET) and small intestinal transit times (SITT) can be determined from the first gastric, duodenal and caecal images [9], whereas colonic (CTT) and whole gut transit times (WGTT) can be determined from the first caecal images and the capsule ingestion and excretion times [10].

WCE systems rely on visible light (white light) to visualise the gut mucosa. IC systems utilising other imaging modalities such as ultrasound (The SonoPill Program [11]) or ionising radiation (The C-Scan® System, Check-Cap Ltd, Isfiya, Israel) are also available. An in-depth review of these non-white light imaging capsule systems has been performed by Cummins et al., [12].

S2.2. pH, temperature and pressure sensing systems

Early pH-sensing capsule systems led to the development of the Wireless Motility Capsule (WMC), (formerly SmartPill Corporation, Buffalo, NY, USA; now supplied by Medtronic, Minneapolis, MN, USA), introduced in 2003 as a multimodal, indigestible, single-use capsule system capable of the simultaneous monitoring of GI pH, temperature and pressure [4]. The capsule, powered by two batteries, contains a solid-state pressure sensor, an ion sensitive field effect transistor pH electrode and a temperature sensor. It also contains a radiofrequency (RF) transmitter and an antenna to transmit the measured signals [13]. An external data receiver with a range of 1.5 metres, records the received signals and also allows a patient to digitally record a diary of symptoms, meals, sleep and bowel movements [14]. After 5 days, data is downloaded off the external data receiver and analysed in specialised software (MotiliGI; previously SmartPill Corporation, Buffalo, NY, USA; now Medtronic, Minneapolis, MN, USA). Abrupt and stereotypical changes in pH as the capsule moves from one gut segment to another are used to determine gut transit times e.g. GET is defined as the time from ingestion to the point in time when there is an abrupt but sustained increase in pH by ≥ 2 units from the gastric pH baseline to pH ≥ 4, which indicates the capsule's passage from the stomach's acidic en-

vironment to the less acidic duodenum [15]. SITT is defined as the time from this point to when there is a sharp drop in pH of ≥ 1.5 units which occurs as the capsule progresses through the ileo-caecal valve into the caecum or ascending colon and at least 30 minutes after the capsule's entry into the small intestine [16]. CTT is then determined from this point to the capsule expulsion time, which along with the capsule ingestion time is determined from sharp changes in the temperature sensor readings. The points in time at which these changes in temperature occur are used to determine WGTT. Single point pressure measurements are made using the WMC, with recording of both amplitude and frequency of gut contractions;[14] however, such measurements have not been seen to be sufficient enough to describe propagating peristaltic contractions and are yet to have an established clinical use [13, 17].

Other applications of gut pH sensing include targeted drug delivery. The IntelliCap System (Medimetrics, Eindhoven, The Netherlands), is one such ingestible drug delivery and monitoring system, that consists of a drug reservoir, pH and temperature sensors, a microprocessor, transceiver for 2-way wireless communication and batteries [18, 19]. Real-time changes in temperature and pH measurements enable localisation of the capsule and therefore enable controlled drug delivery[18].

S2.3. Single-sensor temperature sensing systems

Single-sensor ingestible temperature sensing capsules are also available, with the earliest continuous measurements of core body temperature dating back to the 1960s [20, 21]. Applications of such systems have ranged from understanding thermoregulation and heat stresses induced by exercise [22] or illness [23] to monitoring core body temperatures of astronauts [24]. The CorTemp Monitoring System (HQ Inc., Palmetto, FL, USA), formerly known as the Ingestible Thermal Monitoring System (ITMS) is commercially available for clinical use [25]. The CorTemp capsule is encapsulated in medical-grade epoxy and contains a battery and a temperature-sensitive quartz crystal oscillator, the frequency of which varies with temperature [25, 26]. The measured temperature values are transmitted as a radio signal every 20 seconds to an external data recorder worn around a subject's waist [27]. Other ingestible temperature systems include VitalSense (Philips Respironics, Oregon, USA) [25], e-Celsius performance pill (BodyCap, Caen, France) [28] and myTemp (myTemp, Nijmegen, the Netherlands), which is currently in prototype form [29]. Only WGTT can be obtained from temperature sensing systems. As with pH-sensing systems that incorporate temperature sensors, abrupt changes in temperature when a capsule is ingested and expelled are used to determine WGTT.

S2.4. Magnetic tracking systems

Magnetic and electromagnetic tracking systems are used in a wide range of motion tracking applications so it's not surprising that the use of such systems extended to monitoring GI motility. The earliest use of ingestible magnets to assess GI motility dates back to the late 1950s and early 1960s, where the progression of a permanent magnet through the GI tract was monitored using a magnetometer [17, 30]. Such early studies demonstrated the correlation of a magnet's motion with GI motility in the stomach, which led to the subsequent development of other ingestible magnetic systems to monitor motility in other parts of the gut. Although these systems are mostly confined to the research domain, they have provided some interesting results, such as first images of marker movements in the intestines, which don't require the use of ionising radiation. This was demonstrated in the early 1990s by Weitschies et al., [31] who tracked the progression of magnetically marked drug dosage forms through the GI tract using a 7-channel, superconducting quantum interference device (SQUID) as the detector. To improve visualisation and resolution, a 37-channel SQUID detector was used in a subsequent tracking study with the position of the capsule in the GI tract determined from

the measured field distribution using a localisation algorithm [32]. This Magnetic Marker Monitoring (MMM) system was shown to be accurate, with good temporal and spatial resolution, however it was very complex in set-up and analysis, and required the use of a magnetically-shielded environment.

A simpler, inexpensive system was developed by Andrä et al., [33, 34] which used a permanent magnet, aligning magnetic coils and a magnetic field sensor. The localisation method for this system relies on the measurement of the permanent magnet's magnetic field, which is repeatedly aligned vertically by the magnetic coils, thus generating stray field components that are detected by the magnetic field sensor. These measurements are then used to calculate the magnet's 3-dimensional position in space [33, 34]. The system was simple to use, providing real-time monitoring of GI motility, however like the SQUID system, subjects had to stay relatively immobile in a controlled laboratory environment, with recordings paused to accommodate short breaks for subjects, resulting in non-continuous measurements of gut motility.

The need for an ambulatory system to continuously monitor gut motility was addressed by the 3D-Transit electromagnet tracking system. It continuously tracks and measures the position and orientation of up to three ingestible electromagnetic capsules from ingestion to expulsion using an external detector plate positioned over the abdomen [35]. The original version of the system, the MTS-1 was developed as a stationary, static laboratory-based system using a permanent magnet and a fixed detector plate [36, 37]. However, it was limited to use within a laboratory setting and suffered from interference from large ferromagnetic items and surrounding ambient fields, a disadvantage of using a permanent magnet. To resolve these issues, the ambulatory version uses an electromagnet instead of a permanent magnet, which generates a modulated magnetic field that can be differentiated from the earth's magnetic field, making it easier for the detector to identify and track the electromagnet at any point in space. This removed the need to keep the detector plate fixed in position allowing it to be attached to the body using a belt positioned over the abdomen thereby making the system ambulatory [38]. The electromagnet is encased within a capsule, which also contains an electronic module and a battery. Once activated and swallowed, the electromagnetic capsule emits an electromagnetic tracking signal at a frequency of 5Hz or 10 Hz (pulse rate), which is detected by the detector plate. The frequency of the tracking signal determines the capsule's battery life which ranges between 60 hours (at 10 Hz) and 120 hours (at 5 Hz). During a recording, the electromagnetic tracking signal is saved onto a microSD memory card (Swissbit AG, Switzerland), which is embedded within the detector. Once a recording is complete, the data are downloaded to a computer and converted into 3D-space-time coordinates using dedicated software (Version 0.4-11, Motilis Medica, SA, Lausanne, Switzerland) that uses the detector as a reference point. This enables visualization of a capsule's 3D-position in the gut and its orientation, measured as two angles about the x and y axes. A detailed description of the system and its use in research is provided elsewhere [39].

Magnetic tracking systems rely on the identification of anatomical markers to determine gut transit times, which either take the form of capsule progression pathways, movements and velocities, [40] or in the case of the 3D-Transit electromagnet tracking system, capsule oscillations, which are dictated by the underlying gut contractile activity e.g. GET is defined as the time between capsule ingestion and passage into the duodenum, which is characterised by a drop in capsule oscillation / contractile frequency from 3 contractions per minute (cpm) to 9 – 12 cpm,; SITT is determined as the time from passage into the duodenum to progression of the capsule into the caecum, characterised by a drop in frequency from 9 – 12 cpm to approximately 3 cpm; CTT is then determined from this point in time to the capsule expulsion time and WGTT is simply determined from the capsule ingestion and expulsion times [41].

S2.5. Gas sensing ingestible systems

The latest development in IC systems is the use of gas sensing capsules [42] as an alternative to breath tests, which suffer from inaccuracies due to low gas concentrations [43] and inconsistent interpretation of results [44]. Direct measurement and localisation of gases at the point of production in the GI tract therefore offers potentially better accuracy and reliability of measurements [43, 45]. Earlier developmental versions of the gas sensing capsule contained gas sensors that measured oxygen, hydrogen, carbon dioxide and methane levels in aerobic and anaerobic conditions within the gut [45]. The capsule also contained a temperature sensor, a microcontroller, transmission system and batteries. Gas concentration and temperature readings were transmitted in real-time to a hand-held monitor every 5 minutes, which displayed the gas profiles in real-time on a mobile-phone application via Bluetooth. Prior to ingestion, capsules were calibrated against a range of gas mixtures of known concentrations [45]. Amongst other indications, the measured gas concentrations were used to determine regional gut transit times. However, from early studies, it was recognised that the landmarks identified from the gas profiles to determine GET and SITT were not appropriate as the estimates for GET were too long and those for SITT too short [45, 46].

The sensor technology and software were therefore updated to measure relative humidity, hydrogen and carbon dioxide concentration, along with concentrations of total relative volatile organic compounds, temperature, capsule orientation and changes in the physical electro-magnetic properties of the capsule's environment [46]. The latest version of the capsule therefore uses different measures for landmark assessment which have yielded more consistent estimates. GET is now determined from the time the capsule enters the stomach (indicated by an increase in temperature) to passage from the stomach to the duodenum (i.e. the gastroduodenal junction, identified by an increase in carbon dioxide concentration, changes in capsule orientation and electromagnetic properties of the environment); SITT is determined from the point the capsule enters the duodenum to when it passes from the ileum to the caecum (i.e. the ileocaecal junction, identified when the volatile organic compound sensor conductance changes along with a step change in the electromagnetic properties of the environment). CTT is determined from the ileocaecal junction to capsule excretion, which is identified from a drop in temperature [46].

Section S2 References

1. Iddan, G., et al., *Wireless capsule endoscopy.* Nature, 2000. **405**(6785): p. 417.
2. Swain, P., *Wireless capsule endoscopy.* Gut, 2003. **52 Suppl 4**: p. iv48-50.
3. Van de Bruaene, C., D. De Looze, and P. Hindryckx, *Small bowel capsule endoscopy: Where are we after almost 15 years of use?* World J Gastrointest Endosc, 2015. **7**(1): p. 13-36.
4. Steiger, C., et al., *Ingestible electronics for diagnostics and therapy.* Nature Reviews Materials, 2018.
5. Rey, J.-F., K. Kuznetsov, and E. Vazquez-Ballesteros, *Olympus Capsule Endoscope for Small and Large Bowel Exploration.* Gastrointestinal Endoscopy, 2006. **63**(5): p. AB176.
6. Li, C.Y., et al., *OMOM capsule endoscopy in diagnosis of small bowel disease.* J Zhejiang Univ Sci B, 2008. **9**(11): p. 857-62.
7. Bang, S., et al., *First clinical trial of the "MiRo" capsule endoscope by using a novel transmission technology: electric-field propagation.* Gastrointest Endosc, 2009. **69**(2): p. 253-9.
8. Friedrich, K., et al., *First clinical trial of a newly developed capsule endoscope with panoramic side view for small bowel: a pilot study.* J Gastroenterol Hepatol, 2013. **28**(9): p. 1496-501.
9. Triantafyllou, K., et al., *Video-capsule endoscopy gastric and small bowel transit time and completeness of the examination in patients with diabetes mellitus.* Dig Liver Dis, 2007. **39**(6): p. 575-80.

10. Kastenberg, D., et al., *Multicenter, randomized study to optimize bowel preparation for colon capsule endoscopy.* World journal of gastroenterology, 2017. **23**(48): p. 8615-8625.
11. Cox, B.F., et al., *Ultrasound capsule endoscopy: sounding out the future.* Ann Transl Med, 2017. **5**(9): p. 201.
12. Cummins, G., et al., *Gastrointestinal diagnosis using non-white light imaging capsule endoscopy.* Nature Reviews Gastroenterology & Hepatology, 2019. **16**(7): p. 429-447.
13. Dinning, P.G., et al., *Technical advances in monitoring human motility patterns.* Neurogastroenterol Motil, 2010. **22**(4): p. 366-80.
14. Farmer, A.D., S.M. Scott, and A.R. Hobson, *Gastrointestinal motility revisited: The wireless motility capsule.* United European Gastroenterol J, 2013. **1**(6): p. 413-21.
15. Evans, D.F., et al., *Measurement of gastrointestinal pH profiles in normal ambulant human subjects.* Gut, 1988. **29**(8): p. 1035-41.
16. Zarate, N., et al., *Accurate localization of a fall in pH within the ileocecal region: validation using a dual-scintigraphic technique.* Am J Physiol Gastrointest Liver Physiol, 2010. **299**(6): p. G1276-86.
17. Wenger, M.A., et al., *Stomach Motility In Man As Recorded By The Magnetometer Method.* Gastroenterology, 1961. **41**(5): p. 479-485.
18. van der Schaar, P.J., et al., *First in Human Study With a Novel Ingestible Electronic Drug Delivery and Monitoring Device: The Intellicap.* Gastroenterology, 2011. **140**(5, Supplement 1): p. S-766.
19. Soderlind, E., et al., *Validation of the IntelliCap(R) system as a tool to evaluate extended release profiles in human GI tract using metoprolol as model drug.* J Control Release, 2015. **217**: p. 300-7.
20. Fox, R., R. Goldsmith, and H. Wolff. *USE OF A RADIO PILL TO MEASURE DEEP BODY TEMPERATURE.* in *JOURNAL OF PHYSIOLOGY-LONDON*. 1962. CAMBRIDGE UNIV PRESS 40 WEST 20TH STREET, NEW YORK, NY 10011-4211.
21. MacKay, R.S., *Temperature Sensing and Transmission*, in *Bio-Medical Telemetry: Sensing and Transmitting Biological Information from Animals and Man*. 1993, IEEE. p. 148-160.
22. Byrne, C. and C.L. Lim, *The ingestible telemetric body core temperature sensor: a review of validity and exercise applications.* British journal of sports medicine, 2007. **41**(3): p. 126-133.
23. Huang, F., C. Magnin, and P. Brouqui, *Ingestible sensors correlate closely with peripheral temperature measurements in febrile patients.* J Infect, 2020. **80**(2): p. 161-166.
24. Cutchis, P.N., A.F. Hogrefe, and J.C. Lesho, *The ingestible thermal monitoring system.* Johns Hopkins APL Technical Digest, 1988. **9**(1): p. 16-22.
25. McKenzie, J.E. and D.W. Osgood, *Validation of a new telemetric core temperature monitor.* Journal of Thermal Biology, 2004. **29**(7): p. 605-611.
26. O'Brien, C., et al., *Telemetry pill measurement of core temperature in humans during active heating and cooling.* Med Sci Sports Exerc, 1998. **30**(3): p. 468-72.
27. Monnard, C.R., et al., *Issues in Continuous 24-h Core Body Temperature Monitoring in Humans Using an Ingestible Capsule Telemetric Sensor.* Front Endocrinol (Lausanne), 2017. **8**: p. 130.
28. Travers, G.J.S., et al., *Validation of an ingestible temperature data logging and telemetry system during exercise in the heat.* Temperature (Austin, Tex.), 2016. **3**(2): p. 208-219.
29. Bongers, C.C.W.G., M.T.E. Hopman, and T.M.H. Eijsvogels, *Validity and reliability of the myTemp ingestible temperature capsule.* Journal of Science and Medicine in Sport, 2018. **21**(3): p. 322-326.
30. Wenger, M.A., E.B. Henderson, and J.S. Dinning, *Magnetometer Method for Recording Gastric Motility.* Science, 1957. **125**(3255): p. 990-991.

31. Weitschies, W., et al., *Magnetic markers as a noninvasive tool to monitor gastrointestinal transit.* IEEE Trans Biomed Eng, 1994. **41**(2): p. 192-5.
32. Weitschies, W., et al., *High-resolution monitoring of the gastrointestinal transit of a magnetically marked capsule.* J Pharm Sci, 1997. **86**(11): p. 1218-22.
33. Andra, W., et al., *A novel method for real-time magnetic marker monitoring in the gastrointestinal tract.* Phys Med Biol, 2000. **45**(10): p. 3081-93.
34. Andra, W., et al., *A novel magnetic method for examination of bowel motility.* Med Phys, 2005. **32**(9): p. 2942-4.
35. Gronlund, D., et al., *Established and emerging methods for assessment of small and large intestinal motility.* Neurogastroenterol Motil, 2017. **29**(7).
36. Schlageter, V., et al., *Tracking system with five degrees of freedom using a 2D-array of Hall sensors and a permanent magnet.* Sensors and Actuators A: Physical, 2001. **92**(1): p. 37-42.
37. Schlageter, V., *PhD Thesis: Magnetic tracking system for clinical investigation of gastrointestinal motility*, in *Institute of Microelectronics and Microsystems*. 2003, École polytechnique fédérale de Lausanne: Switzerland. p. 189.
38. Schlageter, V., *Motilis 3D-Transit Gastrointestinal Motility Monitoring System with Software v0.4 Instructions for Use*. 2014, Motilis: Switzerland.
39. Brinck, C.E., et al., *Magnetic tracking of gastrointestinal motility.* Physiol Meas, 2020. **41**(12): p. 12tr01.
40. Weitschies, W., H. Blume, and H. Monnikes, *Magnetic marker monitoring: high resolution real-time tracking of oral solid dosage forms in the gastrointestinal tract.* Eur J Pharm Biopharm, 2010. **74**(1): p. 93-101.
41. Haase, A.M., et al., *Pilot study trialling a new ambulatory method for the clinical assessment of regional gastrointestinal transit using multiple electromagnetic capsules.* Neurogastroenterol Motil, 2014. **26**(12): p. 1783-91.
42. Ou, J.Z., et al., *Human intestinal gas measurement systems: in vitro fermentation and gas capsules.* Trends in Biotechnology, 2015. **33**(4): p. 208-213.
43. Kalantar-Zadeh, K., et al., *Ingestible Sensors.* ACS Sens, 2017. **2**(4): p. 468-483.
44. Simrén, M. and P.O. Stotzer, *Use and abuse of hydrogen breath tests.* Gut, 2006. **55**(3): p. 297-303.
45. Berean, K.J., et al., *The safety and sensitivity of a telemetric capsule to monitor gastrointestinal hydrogen production in vivo in healthy subjects: a pilot trial comparison to concurrent breath analysis.* Aliment Pharmacol Ther, 2018. **48**(6): p. 646-654.
46. Thwaites, P.A., et al., *Comparison of gastrointestinal landmarks using the gas-sensing capsule and wireless motility capsule.* Aliment Pharmacol Ther, 2022. **56**(9): p. 1337-1348.

Section S3: List of studies utilising data from the same healthy volunteer cohorts

Study included in this systematic review:

Wang YT, Mohammed SD, Farmer AD, et al. Regional gastrointestinal transit and pH studied in 215 healthy volunteers using the wireless motility capsule: influence of age, gender, study country and testing protocol. Aliment Pharmacol Ther. 2015;42(6):761-772

Data pooled from healthy volunteers included in studies shown below and reanalysed by Wang et al, 2015:

1. Kuo B, McCallum RW, Koch KL, Sitrin MD, Wo JM, Chey WD, Hasler WL, Lackner JM, Katz LA, Semler JR, Wilding GE, Parkman HP. Comparison of gastric emptying of a nondigestible capsule to a radio-labelled meal in healthy and gastroparetic subjects. Aliment Pharmacol Ther. 2008 Jan 15;27(2):186-96. doi: 10.1111/j.1365-2036.2007.03564.x. Epub 2007 Oct 28. PMID: 17973643.

2. Rao SS, Kuo B, McCallum RW, Chey WD, DiBaise JK, Hasler WL, Koch KL, Lackner JM, Miller C, Saad R, Semler JR, Sitrin MD, Wilding GE, Parkman HP. Investigation of colonic and whole-gut transit with wireless motility capsule and radiopaque markers in constipation. Clin Gastroenterol Hepatol. 2009 May;7(5):537-44. doi: 10.1016/j.cgh.2009.01.017. PMID: 19418602.

3. Sarosiek I, Selover KH, Katz LA, Semler JR, Wilding GE, Lackner JM, Sitrin MD, Kuo B, Chey WD, Hasler WL, Koch KL, Parkman HP, Sarosiek J, McCallum RW. The assessment of regional gut transit times in healthy controls and patients with gastroparesis using wireless motility technology. Aliment Pharmacol Ther. 2010 Jan 15;31(2):313-22. doi: 10.1111/j.1365-2036.2009.04162.x. Epub 2009 Oct 8. PMID: 19814743; PMCID: PMC4444219.

4. Saad RJ, Rao SS, Koch KL, Kuo B, Parkman HP, McCallum RW, Sitrin MD, Wilding GE, Semler JR, Chey WD. Do stool form and frequency correlate with whole-gut and colonic transit? Results from a multicenter study in constipated individuals and healthy controls. Am J Gastroenterol. 2010 Feb;105(2):403-11. doi: 10.1038/ajg.2009.612. Epub 2009 Nov 3. PMID: 19888202.

5. Michalek W, Semler JR, Kuo B. Impact of acid suppression on upper gastrointestinal pH and motility. Dig Dis Sci. 2011 Jun;56(6):1735-42. doi: 10.1007/s10620-010-1479-8. Epub 2010 Nov 18. PMID: 21086166.

6. Rauch S, Krueger K, Turan A, You J, Roewer N, Sessler DI. Use of wireless motility capsule to determine gastric emptying and small intestinal transit times in critically ill trauma patients. J Crit Care. 2012 Oct;27(5):534.e7-12. doi: 10.1016/j.jcrc.2011.12.002. Epub 2012 Feb 1. PMID: 22300488.

Subsequent studies published using either the pooled data from Wang et al. 2015, or from healthy volunteer data contributing to Wang et al. 2015:

1. Farmer AD, Wegeberg AL, Brock B, Hobson AR, Mohammed SD, Scott SM, Bruckner- Holt CE, Semler JR, Hasler WL, Hellström PM, Drewes AM, Brock C. Regional gastrointestinal contractility parameters using the wire-

less motility capsule: inter-observer reproducibility and influence of age, gender and study country. Aliment Pharmacol Ther. 2018 Feb;47(3):391-400. doi: 10.1111/apt.14438. Epub 2017 Dec 6. PMID: 29210098.

2. Wegeberg AL, Brock C, Brock B, Farmer AD, Hobson AR, Semler JR, Scott SM. Regional gastrointestinal pH profile is altered in patients with type 1 diabetes and peripheral neuropathy. Neurogastroenterol Motil. 2018 Nov;30(11):e13407. doi: 10.1111/nmo.13407. Epub 2018 Jul 30. PMID: 30062823.

3. Diaz Tartera HO, Webb DL, Al-Saffar AK, Halim MA, Lindberg G, Sangfelt P, Hellström PM. Validation of SmartPill® wireless motility capsule for gastrointestinal transit time: Intra-subject variability, software accuracy and comparison with video capsule endoscopy. Neurogastroenterol Motil. 2017 Oct;29(10):1-9. doi: 10.1111/nmo.13107. Epub 2017 May 19. PMID: 28524600.

4. Surjanhata B, Brun R, Wilding G, Semler J, Kuo B. Small bowel fed response as measured by wireless motility capsule: Comparative analysis in healthy, gastroparetic, and constipated subjects. Neurogastroenterol Motil. 2018 May;30(5):e13268. doi: 10.1111/nmo.13268. Epub 2017 Dec 18. PMID: 29250864.

5. Surjanhata B, Barshop K, Staller K, Semler J, Guay L, Kuo B. Colonic motor response to wakening is blunted in slow transit constipation as detected by wireless motility capsule. Clin Transl Gastroenterol. 2018 Apr 25;9(4):144. doi: 10.1038/s41424-018-0012-9. PMID: 29691377; PMCID: PMC5915586.

6. Aburub A, Fischer M, Camilleri M, Semler JR, Fadda HM. Comparison of pH and motility of the small intestine of healthy subjects and patients with symptomatic constipation using the wireless motility capsule. Int J Pharm. 2018 Jun 10;544(1):158-164. doi: 10.1016/j.ijpharm.2018.04.031. Epub 2018 Apr 17. PMID: 29678546.

7. Chander Roland B, Garcia-Tsao G, Ciarleglio MM, Deng Y, Sheth A. Decompensated cirrhotics have slower intestinal transit times as compared with compensated cirrhotics and healthy controls. J Clin Gastroenterol. 2013 Nov- Dec;47(10):888-93. doi: 10.1097/MCG.0b013e31829006bb. PMID: 23632359.

8. Abbas A, Wilding GE, Sitrin MD. Does Colonic Transit Time Affect Colonic pH? Journal of Gastroenterology and Hepatology Research 2014; 3(6): 1103-1107 Available from: URL: http://www.ghrnet.org/index.php/joghr/article/view/735

Study included in this systematic review:

Nandhra GK, Mark EB, Di Tanna GL, et al. Normative values for region-specific colonic and gastrointestinal transit times in 111 healthy volunteers using the 3D-Transit electromagnet tracking system: Influence of age, gender, and body mass index. Neurogastroenterol Motil. 2020;32(2):e13734.

Data pooled from healthy volunteers included in studies shown below and reanalysed by Nandhra et al, 2020:

1. Poulsen JL, Nilsson M, Brock C, Sandberg TH, Krogh K, Drewes AM. The Impact of Opioid Treatment on Regional Gastrointestinal Transit. J Neurogastroenterol Motil. 2016 Apr 30;22(2):282-91. doi: 10.5056/jnm15175. PMID: 26811503

2. Mark EB, Poulsen JL, Haase AM, Frøkjaer JB, Schlageter V, Scott SM, Krogh K, Drewes AM. Assessment of colorectal length using the electromagnetic capsule tracking system: a comparative validation study in healthy subjects. Colorectal Dis. 2017 Sep;19(9):O350-O357. doi: 10.1111/codi.13810. PMID: 28688203

3. Poulsen JL, Mark EB, Brock C, Frøkjær JB, Krogh K, Drewes AM Colorectal Transit and Volume During Treatment With Prolonged-release Oxycodone/Naloxone Versus Oxycodone Plus Macrogol 3350. J Neurogastroenterol Motil. 2018 Jan 30;24(1):119-127. doi: 10.5056/jnm17058. PMID: 29291613

4. Kalsi GK, Grønlund D, Martin J, Drewes AM, Scott SM, Birch MJ. Technical report: Inter- and intra-rater reliability of regional gastrointestinal transit times measured using the 3D-Transit electromagnet tracking system. Neurogastroenterol Motil. 2018 Nov;30(11):e13396. doi: 10.1111/nmo.13396. Epub 2018 Jul 4. PMID: 29971879

5. Grønlund D, Vase L, Knudsen SA, Christensen M, Drewes AM, Olesen AE. Comparison of subjective and objective measures of constipation - Employing a new method for categorizing gastrointestinal symptoms. J Pharmacol Toxicol Methods. 2018 Nov-Dec;94(Pt 2):23-28. doi: 10.1016/j.vascn.2018.08.002. Epub 2018 Aug 24. PMID: 30149126 Clinical Trial.

6. Mark EB, Poulsen JL, Haase AM, Espersen M, Gregersen T, Schlageter V, Scott SM, Krogh K, Drewes AM. Ambulatory assessment of colonic motility using the electromagnetic capsule tracking system. Neurogastroenterol Motil. 2019 Feb;31(2):e13451. doi: 10.1111/nmo.13451. Epub 2018 Aug 20. PMID: 30129117

Subsequent studies published using healthy volunteer data contributing to Nandhra et al. 2020:

1. Mark EB, Klinge MW, Grønlund D, Poulsen JL, Schlageter V, Scott SM, Krogh K, Drewes AM. Ambulatory assessment of colonic motility using the electromagnetic capsule tracking system: Effect of opioids.Neurogastroenterol Motil. 2020 Mar;32(3):e13753. doi: 10.1111/nmo.13753. Epub 2019 Nov 12. PMID: 31721398 Clinical Trial.

2. Klinge MW, Haase AM, Mark EB, Sutter N, Fynne LV, Drewes AM, Schlageter V, Lund S, Borghammer P, Krogh K. Colonic motility in patients with type 1 diabetes and gastrointestinal symptoms. Neurogastroenterol Motil. 2020 Dec;32(12):e13948. doi: 10.1111/nmo.13948. Epub 2020 Jul 20. PMID: 32688448

3. Sutter N, Klinge MW, Mark EB, Nandhra G, Haase AM, Poulsen J, Knudsen K, Borghammer P, Schlageter V, Birch M, Scott SM, Drewes AM, Krogh K. Normative values for gastric motility assessed with the 3D-transit electro-

magnetic tracking system. Neurogastroenterol Motil. 2020 Jun;32(6):e13829. doi: 10.1111/nmo.13829. Epub 2020 Mar 10. PMID: 32154975

4. Klinge MW, Sutter N, Mark EB, Haase AM, Borghammer P, Schlageter V, Lund S, Fleischer J, Knudsen K, Drewes AM, Krogh K. Gastric Emptying Time and Volume of the Small Intestine as Objective Markers in Patients With Symptoms of Diabetic Enteropathy. J Neurogastroenterol Motil. 2021 Jul 30;27(3):390-399. doi: 10.5056/jnm19195. PMID: 34210904

Study included in this systematic review:

Sangnes DA, Lundervold K, Bekkelund M, et al. Gastrointestinal transit and contractility in diabetic constipation: A wireless motility capsule study on diabetes patients and healthy controls. United European Gastroenterol J. 2021;9(10):1168-1177.

Used data from the same healthy volunteer cohort (as confirmed in the publication) as:

1. von Volkmann HL, Brønstad I, Gilja OH, R Tronstad R, Sangnes DA, Nortvedt R, Hausken T, Dimcevski G, Fiskerstrand T, Nylund K. Prolonged intestinal transit and diarrhea in patients with an activating GUCY2C mutation. PLoS One. 2017 Sep 28;12(9):e0185496. doi: 10.1371/journal.pone.0185496. PMID: 28957388; PMCID: PMC5619782.

Section S4: Results of Bias Assessment

Table S2: Scores for before-after studies (CD: Cannot determine, N/A: Not applicable)

Criteria	Fujimori, 2010
1. Was the study question or objective clearly stated?	YES
2. Were eligibility/selection criteria for the study population prespecified and clearly described?	NO
3. Were the participants in the study representative of those who would be eligible for the test/service/intervention in the general or clinical population of interest?	NO
4. Were all eligible participants that met the prespecified entry criteria enrolled?	YES
5. Was the sample size sufficiently large to provide confidence in the findings?	NO
6. Was the test/service/intervention clearly described and delivered consistently across the study population?	YES
7. Were the outcome measures prespecified, clearly defined, valid, reliable, and assessed consistently across all study participants?	YES
8. Were the people assessing the outcomes blinded to the participants' exposures/interventions?	YES
9. Was the loss to follow-up after baseline 20% or less? Were those lost to follow-up accounted for in the analysis?	YES
10. Did the statistical methods examine changes in outcome measures from before to after the intervention? Were statistical tests done that provided p values for the pre-to-post changes?	YES
11. Were outcome measures of interest taken multiple times before the intervention and multiple times after the intervention (i.e., did they use an interrupted time-series design)?	NO
12. If the intervention was conducted at a group level (e.g., a whole hospital, a community, etc.) did the statistical analysis take into account the use of individual-level data to determine effects at the group level?	N/A
TOTAL YES	7
TOTAL NO	4
TOTAL CD	0
TOTAL N/A	0
Max no. of questions	11
Total score out max no. of questions	64%

Table S3: Scores for case-series studies (CD: Cannot determine, N/A: Not applicable)

**Criteria**	**Evans, 1988**	**Fallingborg, 1989**	**Hocke, 2009**	**van der Schaar, 2013**	**Haase, 2014**	**Koziolek, 2015**	**O'Grady, 2019**
1. Was the study question or objective clearly stated?	YES	YES	YES	YES	YES	YES	YES
2. Was the study population clearly and fully described, including a case definition?	NO	NO	NO	NO	NO	NO	NO
3. Were the cases consecutive?	NO	NO	YES	YES	NO	CD	YES
4. Were the subjects comparable?	CD	CD	CD	CD	CD	CD	CD
5. Was the intervention clearly described?	YES	YES	YES	YES	YES	YES	YES
6. Were the outcome measures clearly defined, valid, reliable, and implemented consistently across all study participants?	NO	NO	NO	NO	NO	NO	NO
7. Was the length of follow-up adequate?	YES	N/A	NO	YES	YES	NO	YES
8. Were the statistical methods well-described?	YES	NO	YES	NO	YES	NO	YES
9. Were the results well-described?	YES	NO	YES	NO	YES	YES	YES
TOTAL YES	5	2	5	4	5	3	6
TOTAL NO	3	5	3	4	3	4	2
TOTAL CD	1	1	1	1	1	2	1
TOTAL N/A	0	1	0	0	0	0	0
Max no. of questions	8	7	8	8	8	7	8
Total score out max no. of questions	63%	29%	63%	50%	63%	43%	75%

Table S4: Scores for cohort & cross-sectional studies (CD: Cannot determine, N/A: Not applicable)

**Criteria**	**Malagelada, 2008**	**Malagelada, 2012**	**Malagelada, 2015**	**Wang, 2015**	**Monnard, 2017**	**sakurai, 2018**	**Nandhra, 2020**	**Thwaites, 2022**
1. Was the research question or objective in this paper clearly stated?	YES	YES	YES	YES	YES	YES	YES	YES
2. Was the study population clearly specified and defined?	NO	NO	NO	YES	NO	NO	YES	NO
3. Was the participation rate of eligible persons at least 50%?	NO	CD	CD	YES	YES	YES	YES	YES
4. Were all the subjects selected or recruited from the same or similar populations (including the same time period)? Were inclusion and exclusion criteria for being in the study prespecified and applied uniformly to all participants?	NO	NO	CD	NO	CD	YES	NO	NO
5. Was a sample size justification, power description, or variance and effect estimates provided?	NO	NO	YES	NO	NO	NO	NO	YES
6. For the analyses in this paper, were the exposure(s) of interest measured prior to the outcome(s) being measured?	YES	YES	YES	YES	YES	YES	YES	YES
7. Was the timeframe sufficient so that one could reasonably expect to see an association between exposure and outcome if it existed?	CD	YES	YES	YES	CD	CD	CD	CD
8. For exposures that can vary in amount or level, did the study examine different levels of the exposure as related to the outcome (e.g., categories of exposure, or exposure measured as continuous variable)?	YES	YES	YES	N/A	N/A	NO	N/A	NO
9. Were the exposure measures (independent variables) clearly defined, valid, reliable, and implemented consistently across all study participants?	NO	NO	NO	YES	NO	YES	YES	YES
10. Was the exposure(s) assessed more than once over time?	NO	NO	NO	NO	NO	NO	NO	YES
11. Were the outcome measures (dependent variables) clearly defined, valid, reliable, and implemented consistently across all study participants?	YES	NO	YES	YES	YES	YES	YES	YES
12. Were the outcome assessors blinded to the exposure status of participants?	NO	NO	NO	NO	NO	YES	NO	NO

13. Was loss to follow-up after baseline 20% or less?	N/A	N/A	N/A	N/A	N/A	YES	N/A	N/A
14. Were key potential confounding variables measured and adjusted statistically for their impact on the relationship between exposure(s) and outcome(s)?	NO	NO	NO	NO	NO	NO	YES	NO
TOTAL YES	4	4	6	7	4	8	7	7
TOTAL NO	8	8	5	5	6	5	4	5
TOTAL CD	1	1	2	0	2	1	1	1
TOTAL N/A	1	1	1	2	2	0	2	1
Max no. of questions	12	12	11	12	10	13	11	12
Total score out max no. of questions	33%	33%	55%	58%	40%	62%	64%	58%

Table S5: Scores for randomized controlled trial studies (CD: Cannot determine, N/A: Not applicable)

**Criteria**	**Goldstein, 2007**	**Hooks, 2009**	**Jianquin, 2016**	**Mark, 2021**	**Creedon, 2022**
1. Was the study described as randomized, a randomized trial, a randomized clinical trial, or an RCT?	YES	YES	YES	YES	YES
2. Was the method of randomization adequate (i.e., use of randomly generated assignment)?	YES	YES	YES	YES	YES
3. Was the treatment allocation concealed (so that assignments could not be predicted)?	YES	YES	YES	YES	NO
4. Were study participants and providers blinded to treatment group assignment?	YES	YES	YES	YES	NO
5. Were the people assessing the outcomes blinded to the participants' group assignments?	YES	YES	NO	YES	YES
6. Were the groups similar at baseline on important characteristics that could affect outcomes (e.g., demographics, risk factors, co-morbid conditions)?	YES	YES	YES	NO	YES
7. Was the overall drop-out rate from the study at endpoint 20% or lower of the number allocated to treatment?	YES	NO	YES	YES	YES
8. Was the differential drop-out rate (between treatment groups) at endpoint 15 percentage points or lower?	YES	NO	NO	YES	YES
9. Was there high adherence to the intervention protocols for each treatment group?	YES	YES	CD	CD	CD
10. Were other interventions avoided or similar in the groups (e.g., similar background treatments)?	YES	CD	YES	YES	YES
11. Were outcomes assessed using valid and reliable measures, implemented consistently across all study participants?	NO	YES	NO	NO	YES
12. Did the authors report that the sample size was sufficiently large to be able to detect a difference in the main outcome between groups with at least 80% power?	YES	YES	NO	YES	YES
13. Were outcomes reported or subgroups analyzed prespecified (i.e., identified before analyses were conducted)?	YES	YES	YES	YES	YES
14. Were all randomized participants analyzed in the group to which they were originally assigned, i.e., did they use an intention-to-treat analysis?	YES	YES	NO	YES	YES
TOTAL YES	13	11	8	11	11
TOTAL NO	1	2	5	2	2
TOTAL CD	0	1	1	1	1
TOTAL N/A	0	0	0	0	0
Max no. of questions	14	13	13	13	13
Total score out max no. of questions	93%	85%	62%	85%	85%

Table S6: Scores for case-control studies (CD: Cannot determine, N/A: Not applicable)

**Criteria**	**Sangnes, 2021**
1. Was the research question or objective in this paper clearly stated and appropriate?	YES
2. Was the study population clearly specified and defined?	NO
3. Did the authors include a sample size justification?	CD
4. Were controls selected or recruited from the same or similar population that gave rise to the cases (including the same timeframe)?	YES
5. Were the definitions, inclusion and exclusion criteria, algorithms or processes used to identify or select cases and controls valid, reliable, and implemented consistently across all study participants?	YES
6. Were the cases clearly defined and differentiated from controls?	N/A
7. If less than 100 percent of eligible cases and/or controls were selected for the study, were the cases and/or controls randomly selected from those eligible?	NO
8. Was there use of concurrent controls?	YES
9. Were the investigators able to confirm that the exposure/risk occurred prior to the development of the condition or event that defined a participant as a case?	YES
10. Were the measures of exposure/risk clearly defined, valid, reliable, and implemented consistently (including the same time period) across all study participants?	NO
11. Were the assessors of exposure/risk blinded to the case or control status of participants?	NO
12. Were key potential confounding variables measured and adjusted statistically in the analyses? If matching was used, did the investigators account for matching during study analysis?	YES
TOTAL YES	6
TOTAL NO	4
TOTAL CD	1
TOTAL N/A	1
Max no. of questions	10
Total score out max no. of questions	60%

Figure S1: Bias assessment scores

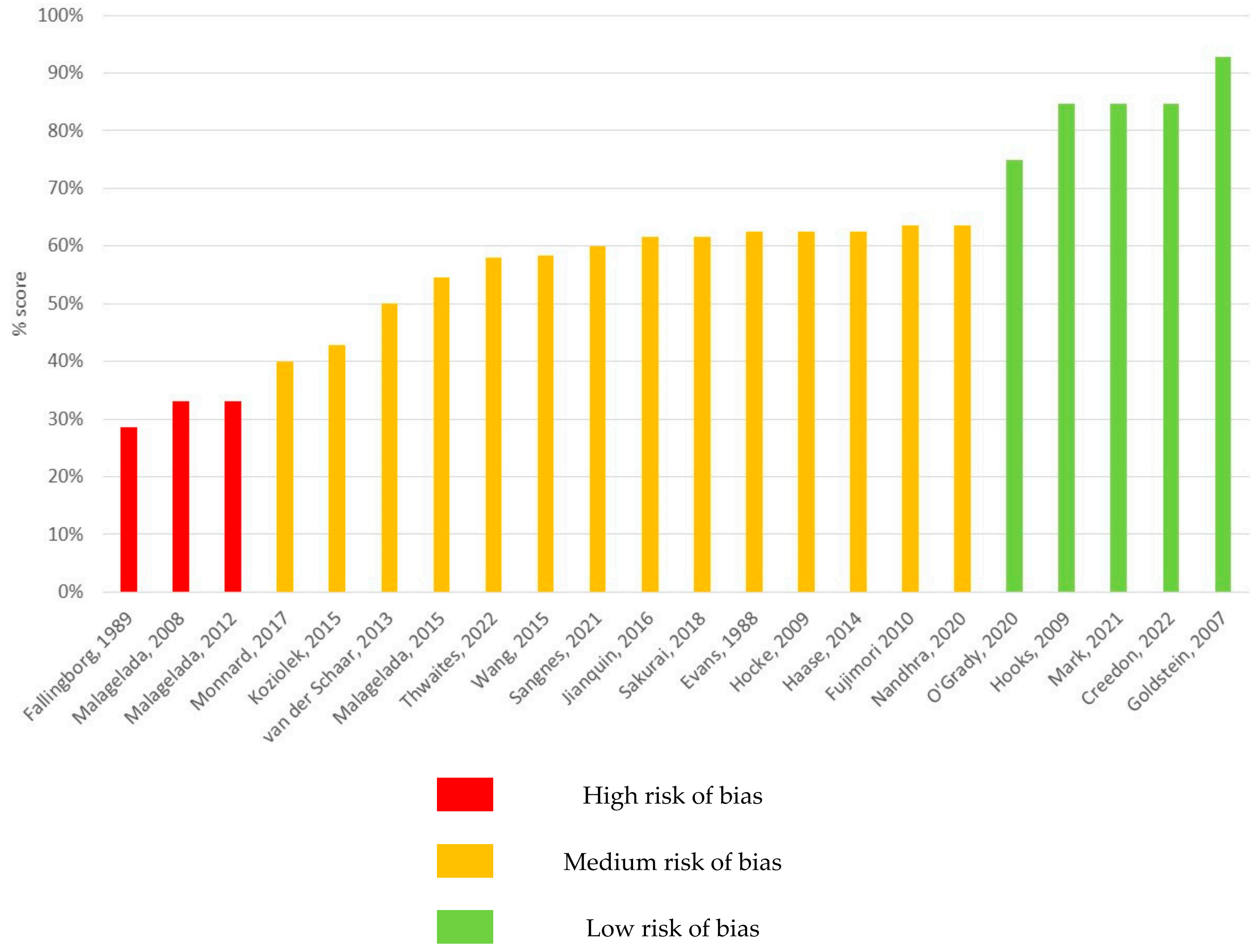

Supplement: Supplementary file 1 [file jcm-12-05272-s001.zip › jcm-2527378-supplementary.pdf]
